# AnnoPRO: a strategy for protein function annotation based on multi-scale protein representation and a hybrid deep learning of dual-path encoding

**DOI:** 10.1186/s13059-024-03166-1

**Published:** 2024-02-01

**Authors:** Lingyan Zheng, Shuiyang Shi, Mingkun Lu, Pan Fang, Ziqi Pan, Hongning Zhang, Zhimeng Zhou, Hanyu Zhang, Minjie Mou, Shijie Huang, Lin Tao, Weiqi Xia, Honglin Li, Zhenyu Zeng, Shun Zhang, Yuzong Chen, Zhaorong Li, Feng Zhu

**Affiliations:** 1 College of Pharmaceutical Sciences, The Second Affiliated Hospital, Zhejiang University School of Medicine, Zhejiang University, Hangzhou, 310058 China; 2 Industry Solutions Research and Development, Alibaba Cloud Computing, Hangzhou, 330110 China; 3 Innovation Institute for Artificial Intelligence in Medicine of Zhejiang University, Alibaba-Zhejiang University Joint Research Center of Future Digital Healthcare, Hangzhou, 330110 China; 4 Key Laboratory of Elemene Class Anti-Cancer Chinese Medicines, Engineering Laboratory of Development and Application of Traditional Chinese Medicines, Collaborative Innovation Center of Traditional Chinese Medicines of Zhejiang Province, School of Pharmacy, Hangzhou Normal University, Hangzhou, 311121 China; 5 Pharmaceutical Department, Zhejiang Provincial People’s Hospital, Hangzhou, 310014 China; 6 School of Pharmacy, East China University of Science and Technology, Shanghai, 200237 China; 7 State Key Laboratory of Chemical Oncogenomics, Key Laboratory of Chemical Biology, The Graduate School at Shenzhen, Tsinghua University, Shenzhen, 518055 China

**Keywords:** Protein function annotation, Long-tail problem, Protein representation, Pre-training, LSTM

## Abstract

Protein function annotation has been one of the longstanding issues in biological sciences, and various computational methods have been developed. However, the existing methods suffer from a serious long-tail problem, with a large number of GO families containing few annotated proteins. Herein, an innovative strategy named AnnoPRO was therefore constructed by enabling sequence-based multi-scale protein representation, dual-path protein encoding using pre-training, and function annotation by long short-term memory-based decoding. A variety of case studies based on different benchmarks were conducted, which confirmed the superior performance of AnnoPRO among available methods. Source code and models have been made freely available at: https://github.com/idrblab/AnnoPRO and https://zenodo.org/records/10012272

**Supplementary Information:**

The online version contains supplementary material available at 10.1186/s13059-024-03166-1.

## Background

Protein function annotation has been one of the longstanding issues, which is key for discovering new drug target and understanding physiological or pathological process [1–3]. With the advance of next-generation sequencing, a large amount of protein sequences have been accumulated, and over 200 million sequences have been available in UniProt [4]. Compared with the accumulation of protein sequences, the experimental annotation of protein functions is much more challenging, which is characterized by its natures of time-consuming and labor-intensive [5–7]. So far, only a very small portion of protein sequences have been successfully annotated based on experimental evidence [4], which asks for the discovery of innovative strategy to greatly accelerate the process of annotation [8]. Thus, many computational methods are developed to facilitate the progress of this field [9–11], which extensively promote the identification of efficacious drug target [12], the revealing of molecular mechanism underlying sophisticated disease etiology [13], and so on.

**Fig. 1:**
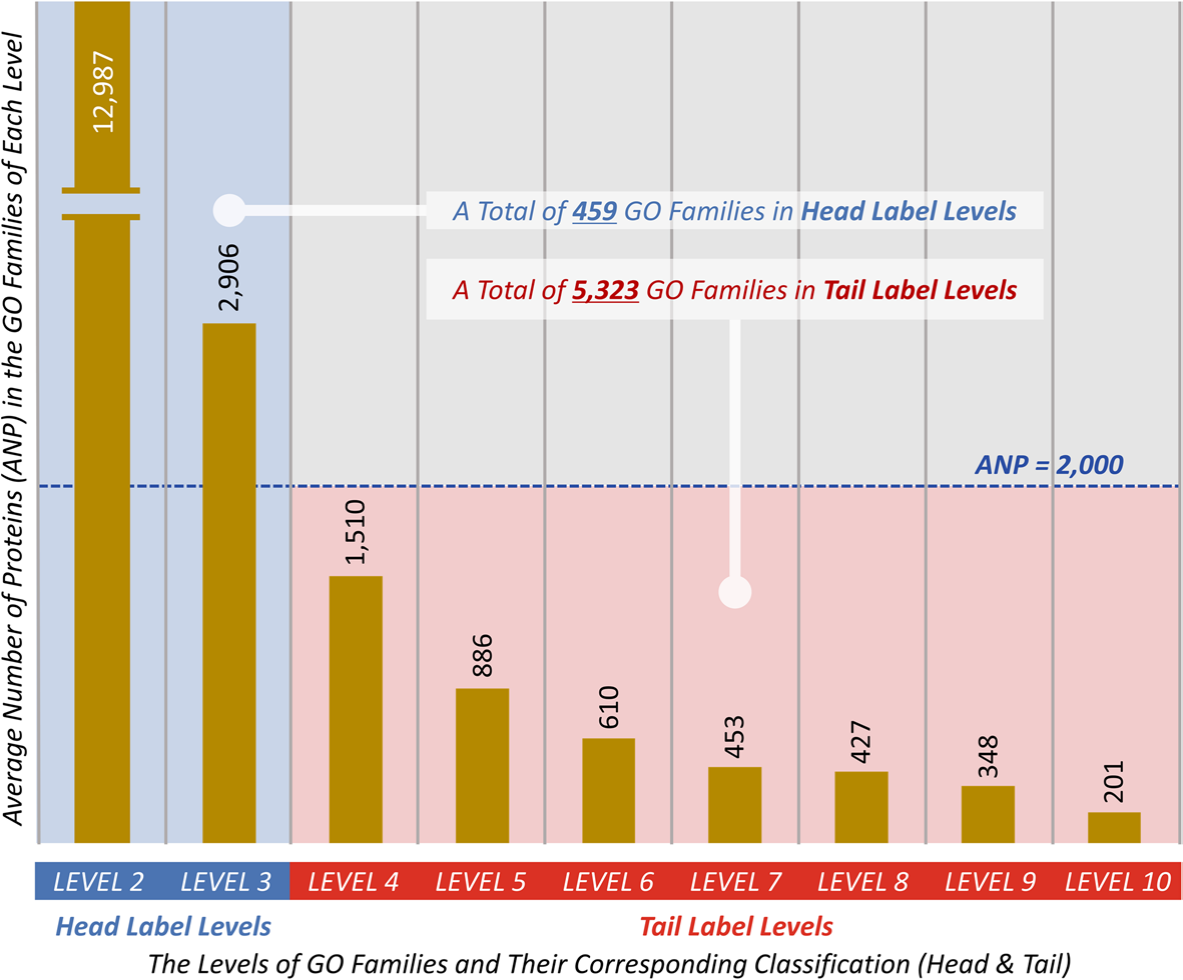
Average number of proteins (ANP) in the GO families of nine different levels (LEVEL 2 to LEVEL 10 as shown in Additional file 1: Fig. S3). There was a clear descending trend of ANPs from the top level (LEVEL 2) to the bottom one (LEVEL 10). Since the ANP of one family indicated its representativeness among all families, this figure denoted a gradual decrease of the representativeness of a family with the penetration into deeper level. Therefore, the nine levels could be classified into two groups based on their ANPs: the “*Head Label Levels*” (ANP of their GO families ≥ 2,000) and the “*Tail Label Levels*” (ANP of their GO families < 2,000). As shown, the total number (5,323) of GO families in the “*Tail Label Levels*” was > 10 times larger than that (459) of the “*Head Label Levels*”, and such kind of data distribution induced a serious ‘*long-tail problem*’ as described in the previous pioneering publication [18]

However, the annotation of protein function using computational method has been suffering from a serious “*long-tail problem*” [14–16] with a large number of functional families containing few annotated proteins. These families are categorized to the ones of “*Tail Label Levels*”, while the remaining are to “*Head Label Levels*”. Based on the current *Gene Ontology* (GO) database [17], the average numbers of proteins (ANP) in those GO families (terms) of different GO levels were assessed and statistically described in Fig. 1, and the number 2,000 was set as a cutoff of ANP for differentiating ‘*Tail Label Levels*’ from ‘*Head Label Levels*’. As shown in Fig. 1, the total number (5,323) of GO families in ‘*Tail Label Levels*’ is more than 10 times larger than that (459) of ‘*Head Label Levels*’ [17]. In other words, the protein functional data in GO database follow a *long-tailed distribution* where only a few ‘*head label*’ families and many ‘*tail label*’ ones present [17]. The ‘*long-tailed phenomenon*’ has been reported to lead to severe degradation of annotation performances due to the serious imbalance problem between the data of *head* and *tail* [18]. This is also the principal reason for *head label* families dominating the training process, making these families enjoy substantially higher accuracies than those *tail label* ones [18–20]. So far, two types of protein function annotation strategy have been constructed, which can be roughly divided into the sequence homology (SH) based ones and the machine learning (ML) based ones [21].

SH-based strategy has long been used for protein function annotations [22], and many tools have been constructed (such as *BLAST* and *GoFDR*) [23, 24], but the accuracy of sequence alignments drops off rapidly in cases where the sequence identity/homology falls below certain critical point [25]. To deal with this issue, ML-based strategy has been proposed, which learns protein function irrespective of sequence homology [26–31], including *DeepGOPlus*, *PFmulDL* and *NetGO2* [14–16]. These tools apply machine learning frameworks to achieve good protein annotation, such as *NetGO2* in “*4th critical assessment of functional annotation*” (CAFA4) challenge [16]. However, due to the overwhelming domination of proteins in the ‘*Head Label Levels*’ (the average number of proteins in the family of *Head Label Levels* equals to 4,210, which is about 5 times larger than that (886) of *Tail Label Levels*, as shown in Fig. 1), it is still extremely challenging for existing methods/tools to improve the prediction accuracies for the families in *Tail Label Levels*, and the “*long-tail problem*” in protein functional annotation remains unsolved [32].

Herein, an innovative strategy, entitled ‘*AnnoPRO*’, for protein function annotation was therefore constructed. *First*, a sequence-based multi-scale protein representation enabling the conversions of protein sequences to both *feature similarity*-based images and *protein similarity*-based vectors was proposed. This representation is unique in not only capturing the intrinsic correlation among protein features, but also taking the global relevance among protein sequences into consideration, which can favor the applications of some deep learning strategies popular in image classification. *Second*, a hybrid deep learning framework of dual-path encoding was constructed for annotating the protein function. Since this framework was inspired, in part, by a method [33] used for image classification to cope with ‘*long-tail problem*’, *AnnoPRO* was expected to significantly improve the annotation performance for the GO families in the ‘*Tail Label Levels*’. *Finally*, multiple case studies using many benchmark datasets were conducted, which further confirmed the superiority of our new strategy among the existing ones. All in all, the *AnnoPRO* performed well and would become an essential complement to existing methods in protein function prediction.

## Results and discussion

### A new hybrid deep learning framework for protein function annotation

**Fig. 2:**
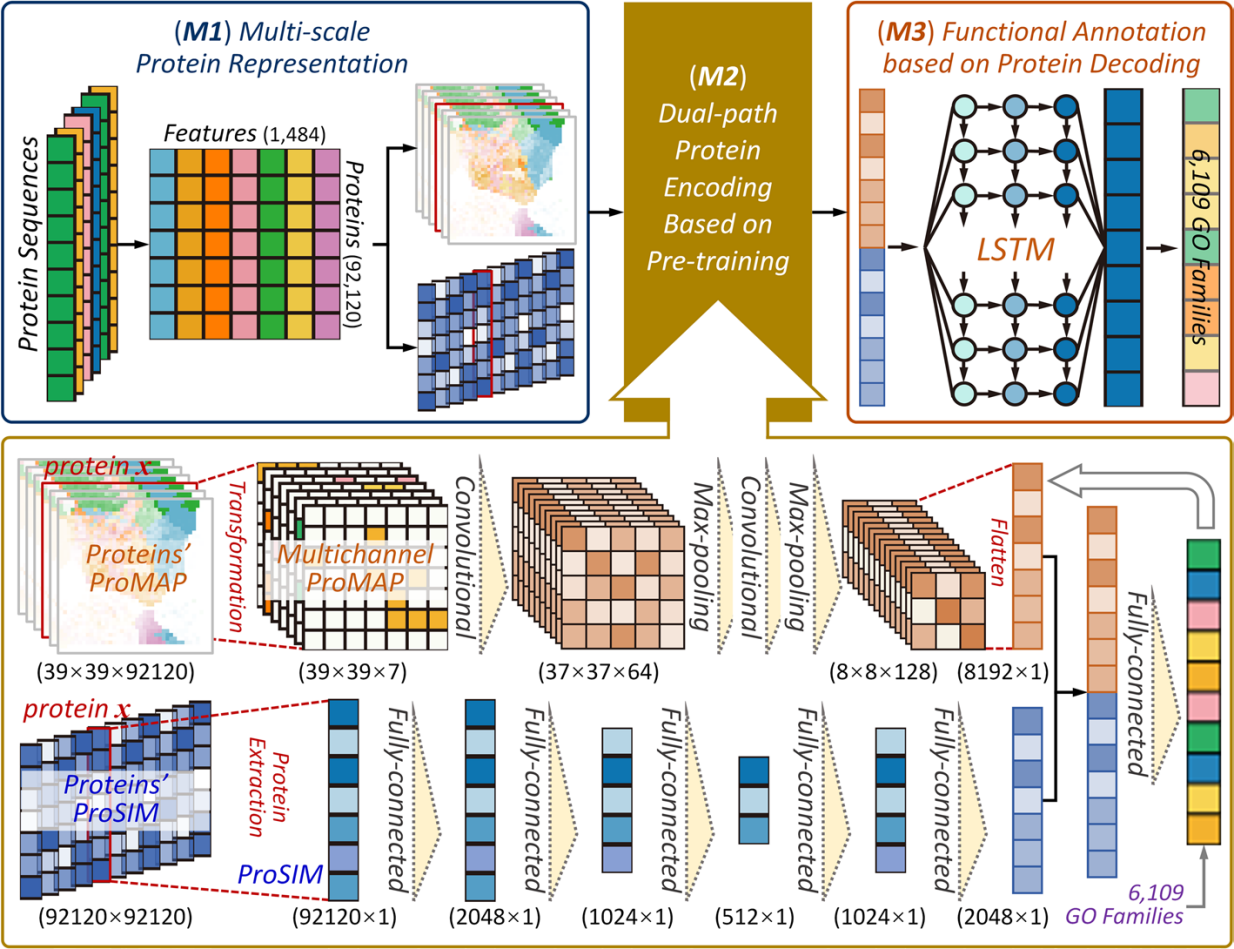
The hybrid deep learning framework of three consecutive modules (*M1* to *M3*) adopted in this study. (*M1*) the sequence-based multi-scale protein representation realizing conversion of all protein sequences to *feature similarity*-based images (*ProMAP*) and *protein similarity*-based vectors (*ProSIM*). (*M2*) the dual-path protein encoding based on pre-training. Using the *ProMAP* and *ProSIM* generated for all the sequences, a dual-path encoding strategy was constructed based on a seven-channel *Convolutional Neural Network* (7C-CNN) and *Deep Neural Network* of five fully-connected layers (5FC-DNN) to pre-train the features of all CAFA4 proteins by integrating their annotation data of GO families. (*M3*) the functional annotation by a LSTM-based decoding. The protein features pre-trained using the dual-path encoding layer in *M2* were concatenated and then fed into a *long short-term memory recurrent neural network* (LSTM) to enable a multi-label annotation of proteins to 6,109 functional GO families using the hybrid deep learning

Herein, a hybrid deep learning framework was constructed to enable protein function annotations, which consisted of three consecutive modules (*M1* to *M3*). As shown in Fig. 2, these modules included: (*M1*) the sequence-based multi-scale protein representation realizing the conversion of all protein sequences to *feature similarity*-based images (*ProMAP*) and *protein similarity*-based vectors (*ProSIM*). Particularly, at *feature similarity* scale, the similarities among protein features were utilized to transform the ‘unordered’ vector of 1,484 protein features to an ‘ordered’ image-like representation; at *protein similarity* scale, the pair-wise similarities between any two proteins were used to transform the ‘independent’ vector of 1,484 protein features to a ‘globally-relevant’ vector of 92,120 dimensions. (*M2*) the dual-path protein encoding based on a pre-training. Using the *ProMAP* and *ProSIM* generated for all proteins, a dual-path encoding was constructed based on a seven-channel *Convolutional Neural Network* (7C-CNN) and *Deep Neural Network* of five fully-connected layers (5FC-DNN) to pre-train the features of all CAFA4 proteins by integrating their annotation data of GO families. (*M3*) the functional annotation by a LSTM-based decoding. The protein features pre-trained using the dual-path encoding layer in *M2* were concatenated and then fed into a *long short-term memory recurrent neural network* (LSTM) to enable a multi-label annotation of proteins to 6,109 functional GO families using the hybrid deep learning. The details of this hybrid deep learning framework were further elaborated in Materials and Methods.

**Fig. 3:**
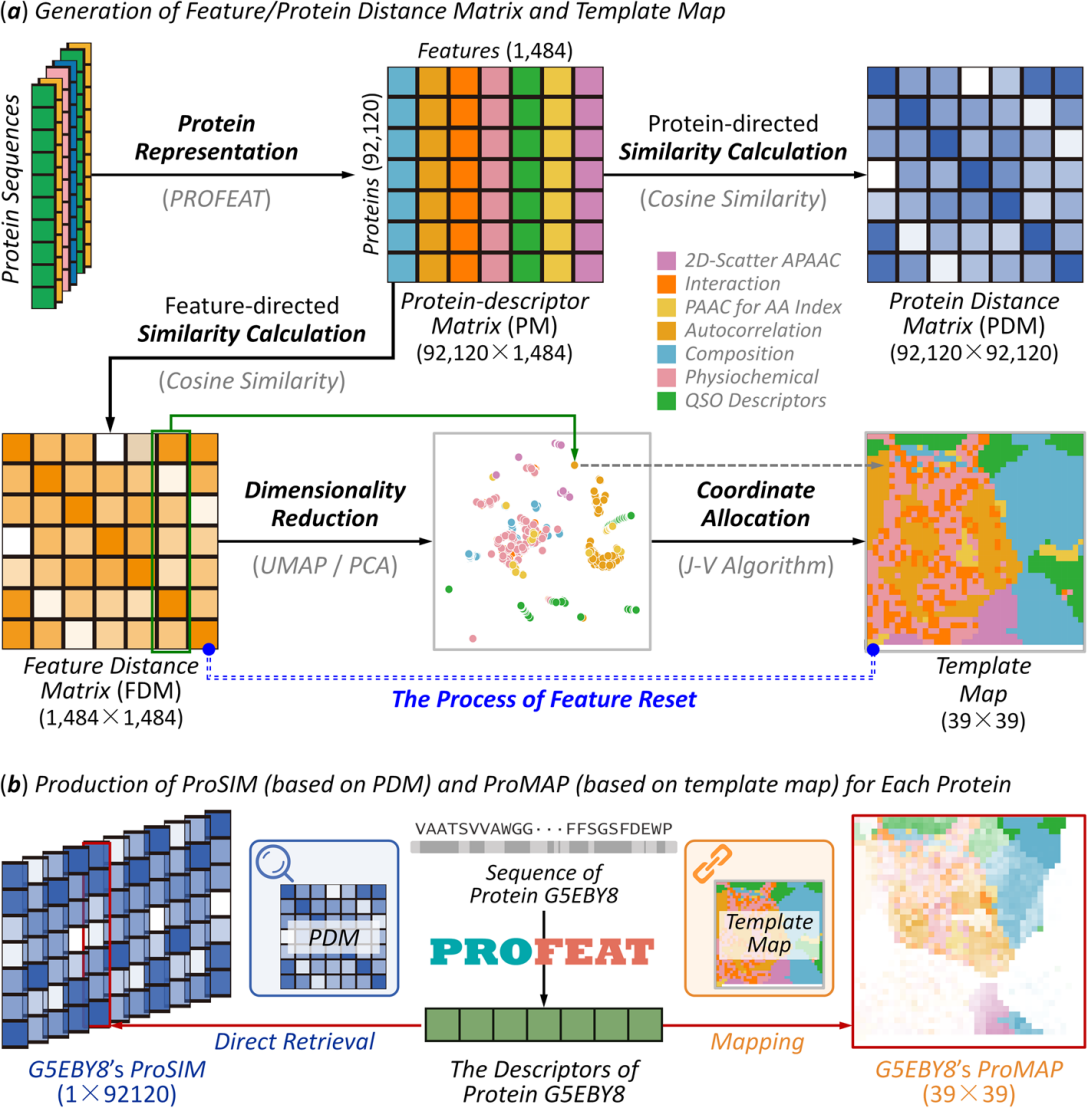
A schematic illustration of the procedure used in this study facilitating sequence-based multi-scale protein representation. The way how sequences were converted to *feature similarity*-based image (*ProMAP*) and *protein similarity*-based vector (*ProSIM*) was shown. (**a**) generation of feature/protein distance matrix and ‘*template map*’; (**b**) production of *ProSIM* (based on PDM) and *ProMAP* (based on *template map*) for each protein. On the one hand, a method realizing the image-like protein representation was constructed (*ProMAP*) to capture the intrinsic correlations among protein features. As illustrated, a *template map* for each protein was *first* constructed by a consecutive process of ‘*protein representation*’ using PROFEAT, ‘*similarity calculation*’ using cosine similarity, ‘*dimensionality reduction*’ using UMAP or PCA, ‘*coordinate allocation*’ using *Jonker-Volgenant algorithm*, etc*. Then*, *ProMAP* was produced for each protein by mapping the intensities of all protein features to their corresponding locations in the constructed *template map* (illustrated on the right side of Fig. 3b). On the other hand, an approach considering the global relevance among proteins was proposed (*ProSIM*) to convert ‘independent’ vector to a ‘globally-relevant’ protein representation. As shown, a *protein distance matrix* (PDM) was first generated by following the consecutive process of ‘*protein representation*’ using PROFEAT and ‘*similarity calculation*’ using cosine similarity. Finally, *ProSIM* was generated for each protein by retrieving directly from each row of the newly generated PDM (shown in the left side of Fig. 3b)

In the hybrid deep leaning framework, the sequence-based multi-scale protein representation was one of the key modules (*M1*). As shown in Fig. 3, the way how the conversion of all sequences to *feature similarity*-based images (*ProMAP*) and *protein similarity*-based vectors (*ProSIM*) was described. On the one hand, a method realizing image-like protein representations was proposed (*ProMAP*) for capturing the intrinsic correlations among protein features. As illustrated in Fig. 3a, a template map for each protein sequence was *first* constructed by a consecutive process of ‘*protein representation*’ (using PROFEAT [34]), ‘*similarity calculation*’ (using *Cosine Similarity* [35]), ‘*dimensionality reduction*’ (using UMAP [36] or PCA [37]), ‘*coordinate allocation*’ (using *Jonker-Volgenant algorithm* [38]), etc*. Then*, *ProMAP* was produced for each protein by mapping the intensities of all protein features to their corresponding locations in the constructed template map (illustrated on the right side of Fig. 3b). On the other hand, an approach considering the global relevance among proteins was proposed (*ProSIM*) to convert the ‘independent’ vector to a ‘globally-relevant’ protein representation. As illustrated in Fig. 3a, a protein distance matrix (PDM) was *first* generated by following a consecutive process of ‘*protein representation*’ (using PROFEAT [34]) and ‘*similarity calculation*’ (using *Cosine Similarity* [35]). *Finally*, *ProSIM* was generated for each protein by retrieving directly from each row of the newly generated PDM (as shown in the left side of Fig. 3b). All in all, these newly proposed strategies could capture the intrinsic correlation among protein features and consider the global relevance among sequences. The detailed description was explicitly provided in the Materials and Methods.

### Comparing the overall performances among *AnnoPRO* and existing tools

In this study, a total of 92,120 protein sequences were *first* collected from the competition of ‘*4th critical assessment of functional annotation*’ (CAFA4, released on Oct 21, 2019) [20], and these data were adopted to construct the annotation model (*Training* and *Validation*). *Second*, a process identical to that of ‘CAFA4’ for constructing the “*Independent Testing Dataset*” was used, which led to a total of 5,623 *SwissProt* proteins [4] with experimentally-validated functional annotation between Oct 22, 2019 and May 31, 2022. As reported, such methodology above for data partition had been frequently adopted by previous studies [14, 16, 39] to develop the functional annotation models and realizing the systematic comparison among existing methods/tools.

To assess the overall performance of our new strategy, a comparison among the performances of *AnnoPRO* and eight popular methods (such as: *Diamond*_*BLAST*_ [24], *DeepGO* [40], *DeepGOCNN* [14], *DeepGOPlus* [14], *TALE* [41], *PFmulDL* [15], *NetGO2* [16], *NetGO3* [31]) was conducted. The strategies of these methods to partition data had been described in the above paragraph, and their processes of model construction were illustrated in Supplementary Method S1. As shown in Table 1, among those eight popular methods, *DeepGOPlus*, *PFmulDL*, and *NetGO3* gave the best performances on the GO data of BP, CC, and MF, respectively (highlighted by the *underline*. *Diamond*_*BLAST*_ provided a better F_*max*_ than *NetGO3* on MF, but its AUPRC was much lower than that of *NetGO3*, thus *NetGO3* was considered to have the best performance on MF). These results showed that there was no existing tool performing consistently the best under all GO classes (BP, CC, and MF). However, as shown in Table 1, comparing with other methods, *AnnoPRO* provided the best performance (highlighted in BOLD) under all GO classes. Particularly, when comparing with the three best performing methods (*DeepGOPlus*, *PFmulDL*, and *NetGO3*), the percentages of performance enhancement varied from 2.7% to 15.7% (as assessed by F_*max*_) and from 2.3% to 22.2% (as assessed by AUPRC), which illustrated a dramatical elevation in the performances of protein functional prediction by the new *AnnoPRO* strategy proposed in this study.

**Table 1:** A comparison among the performances of *AnnoPRO* and eight available methods/tools

Method/Tool	Date of Publication	BP		CC		MF	
		**F**_***max***_	**AUPRC**	**F**_***max***_	**AUPRC**	**F**_***max***_	**AUPRC**
*Diamond*_*BLAST*_	Nov, 2014	0.549	0.183	0.550	0.186	0.729	0.112
*DeepGO*	Feb, 2018	0.362	0.213	0.501	0.434	0.384	0.325
*DeepGOCNN*	Jan, 2020	0.369	0.294	0.516	0.460	0.382	0.362
*DeepGOPlus*	Jan, 2020	0.593	0.561	0.588	0.502	0.628	0.627
*TALE*	Mar, 2021	0.391	0.307	0.562	0.587	0.472	0.458
NetGO2*	Jul, 2021	0.497	0.434	0.574	0.508	0.667	0.674
*PFmulDL*	Mar, 2022	0.324	0.257	0.590	0.608	0.412	0.371
NetGO3*	Dec, 2022	0.540	0.500	0.579	0.535	0.687	0.726
*AnnoPRO*	This Study	**0.609**	**0.574**	**0.746**	**0.749**	**0.763**	**0.755**

The values indicating the best performances among all methods/tools were highlighted in BOLD, and *AnnoPRO* performed consistently the best in all Gene Ontology (GO) classes (BP, CC, MF) under both evaluating criteria (F_*max*_, AUPRC). All methods/tools were ordered according to their publication dates. BP: *biological process*; CC: *cellular component*; MF: *molecular function*; F_*max*_: *protein centric maximum F-measure*; AUPRC: *area under the precision-recall curve*. The tools marked by an asterisk (*) indicated that their source-codes for model construction were not fully provided, which made it impossible for us to train models on experimental functional annotations that appeared before Oct 22, 2019, and their performances (evaluated by F_*max*_ and AUPRC) were assessed by directly uploading those experimental function annotations asserted between Oct 22, 2019 and May 31, 2022 to the online server of those annotation tools. Among those eight existing methods/tools, the best performing ones under each category were highlighted by *underline*

To have an in-depth understanding on the significant elevation in the annotation performance of *AnnoPRO*, an *ablation* experiment [42] was further conducted to assess the performance changes induced by depriving some key *AnnoPRO* modules. As described in Additional file 1: Fig. S1, “No *ProMAP*” indicated that seven-channel *convolutional neural network* (7C-CNN) was made absent from the *Module 2* in Fig. 2, and “No *ProSIM*” presented that *deep neural network* of five fully-connected layers (5FC-DNN) was deprived from the *Module 2* in Fig. 2. As shown, both strategies (*ProMAP* and *ProSIM*) adopted in this study for multi-scale protein representation contributed substantially to the performances of *AnnoPRO* (13.6 ~ 24.2% for AUPRC; 4.6 ~ 22.4% for F_*max*_). On the one hand, *ProMAP* facilitated the discovery of the intrinsic correlations among protein features by transforming the ‘unordered’ vector to an ‘ordered’ image-like representation. On the other hand, *ProSIM* took the global relevance among protein sequences into consideration by converting the ‘independent’ vector to a ‘globally-relevant’ protein representation. Moreover, as shown in Additional file 1: Fig. S1, “No LSTM” represented that *Long Short-Term Memory recurrent neural network* was removed from *Module 3* in Fig. 2, and “SC map” denoted that “*Transformation*” step in *Module 2* in Fig. 2 was deprived and only single-channel (not multi-channel) *ProMAP* was considered. In conclusion, it is clear to see that the deprivation of any key module will result in significant decrease in the annotation performance, which indicated that all the key modules collectively contributed to the good performance of *AnnoPRO*.

When realizing the image-like protein representation (as illustrated in Fig. 3), there were two methods applied for ‘*dimensionality reduction*’, which included *uniform manifold approximation and projection* (UMAP) [36] and *principal component analysis* (PCA) [37]. UMAP was reported to produce arbitrary shapes and distort distances in 2D, which could be severely biased and lead to misinterpretation [43]. Since the image-like protein representation was novel and essential for *AnnoPRO*, it is needed to assess the contributions of UMAP and PCA to annotation performances. Herein, two models *AnnoPRO*_*UMAP*_ and *AnnoPRO*_*PCA*_ were thus constructed based on UMAP and PCA, respectively (the procedure for model construction and evaluation is described above). As shown in Additional file 1: Fig. S2, the performances (assessed by F_*max*_ and AUPRC) of these models are roughly the same across three GO classes (BP, CC, MF). Particularly, *AnnoPRO*_*UMAP*_ showed a slightly better predictive performance compared with *AnnoPRO*_*PCA*_ (0.6 ~ 1.9% for F_*max*_; 1.4 ~ 2.1% for AUPRC). All in all, although concerns were raised about the limitations of UMAP [43], the performance evaluation conducted in this study showed that the application of different dimensionality reduction methods (UMAP *vs* PCA) might not significantly alter the performance. Therefore, both methods (UMAP and PCA) were integrated into the *AnnoPRO* software package (https://pypi.org/project/annopro/0.1rc2/) and the online server (https://idrblab.org/annopro/).

### Level-based performance comparison among *AnnoPRO* and existing tools

**Fig. 4:**
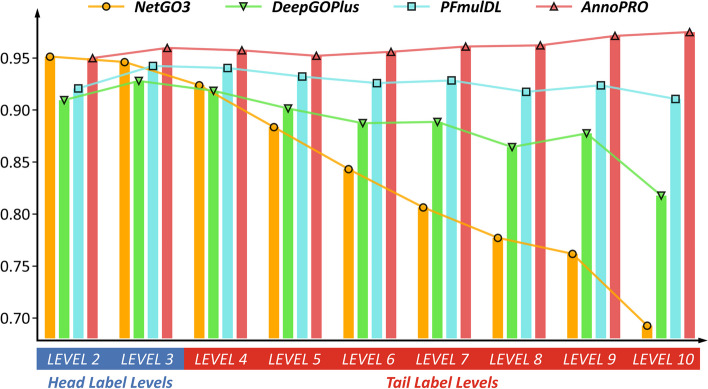
A comparison among the performances of *AnnoPRO* and three representative methods. The performances were represented using AUC values in predicting the experimentally validated new protein functions that were not included in CAFA4 data, and the performances of *AnnoPRO*, *DeepGOPlus*, *NetGO3* and *PFmulDL* were highlighted in light red, light green, orange and light blue, respectively. For GO families in the ‘*Head Label Levels*’ (LEVEL 2 and LEVEL 3 provided in Additional file 1: Fig. S3), the performance of *AnnoPRO* was roughly as good as that of the other three methods (1.4 ~ 4.1% improvements in most cases, but 0.1% decline in one single case). For the GO families in the ‘*Tail Label Levels*’ (LEVEL 4 to LEVEL 10 shown in Additional file 1: Fig. S3), *AnnoPRO* demonstrated the consistently superior performance among four methods (1.7 ~ 28.2% improvements in all cases). Particularly, 13 (61.9%) out of all 21 improvements were over 5%, and 6 (28.6%) out of 21 improvements were more than 10%. Therefore, *AnnoPRO* was identified *superior* in significantly improving the annotation performances of the families in ‘*Tail Label Levels*’ without sacrificing that of the ‘*Head Label Levels*’, which was highly expected to make contribution to solving the long-standing ‘*long-tail problem*’[18] in functional annotation

Based on those analyses above, three recently-published methods (*DeepGOPlus*, *PFmulDL*, and *NetGO3*) were found to perform better than others and reported as “*state-of-the-art*” by previous publication [44]. Therefore, a comparison among the level-based performances of *AnnoPRO* and these SOTA *methods* was conducted. The so-called level-based performances were based on the hierarchical GO families shown in the first section of Materials and Methods and the definition in Additional file 1: Fig. S3. As shown in Fig. 4, the level-based performances were given using AUC value in predicting the testing data, and the performances of *AnnoPRO*, *DeepGOPlus*, *NetGO3*, and *PFmulDL* were shown by light red, light green, orange, and light blue, respectively (also provided in Supplementary Table S1). For GO families in ‘*Head Label Level*’ (LEVEL 2 and LEVEL 3 in Additional file 1: Fig. S3), the performance of *AnnoPRO* was as good as that of other methods (1.4 ~ 4.1% improvements in most cases, but 0.1% decline in one case). For GO families in ‘*Tail Label Level*’ (LEVEL 4 to LEVEL 10 in Additional file 1: Fig. S3), *AnnoPRO* provided the consistently superior performance among all methods (1.7 ~ 28.2% improvement in all cases). Particularly, 13 (61.9%) out of all 21 improvements were over 5%, and 6 (28.6%) out of those 21 improvements were larger than 10% (as illustrated in Fig. 4).

Furthermore, as illustrated in Fig. 4, *DeepGOPlus* and *NetGO3* performed well in LEVEL 2 and LEVEL 3, but experienced a dramatic decline of performance from LEVEL 4 to LEVEL 10. This clearly showed that the “*long tail problem*” remained a serious issue for the protein function annotation using existing methods (significantly declined from 95.1% to 69.3% for *NetGO3* and from 91.8% to 81.8% for *DeepGOPlus*). The *PFmulDL* was a method that could largely enhance the performances for the GO families in ‘*Tail Label Level*’, but *AnnoPRO* provided a much better performances in all levels than *PFmulDL* (as shown in Fig. 4). In other words, *AnnoPRO* was the first method reported to achieve *superior* performance in protein annotations for GO families in ‘*Tail Label*’ levels without sacrificing that in ‘*Head Label*’ ones, which was therefore expected to highly contribute to the final solution of the long-standing ‘*long-tail problem*’.

### Performance comparison based on the proteins from a variety of species

Sequence variation among the orthologs of various species may induce subtle, or even substantial, changes in protein structure, which may lead to proteins with similar sequence showing different functions [45–47]. This leads to great difficulty in functional annotations for orthologous proteins [48], and it is therefore of great interests to compare the capacities of *AnnoPRO* and the *state-of-the-art* methods/tools (*DeepGOPlus*, *PFmulDL* and *NetGO3*) from this perspective. In this study, the species origins of 92,120 proteins from CAFA4 (adopted as ‘*Training*’ and ‘*Validation*’) were *first* assessed, and 17 species were found (*homo sapiens*, *mus musculus*, *drosophila melanogaster*, etc*.*). In the meantime, the species origins of 5,623 proteins (used as ‘*Independent Testing*’) were also found, which discovered a total of 1,014 species (despite those 17 species, there were many other species: *bos taurus*, *camellia sinensis*, *canis lupus familiaris*, *gallus gallus*, *mycobacterium tuberculosis*, *oryza sativa*, etc*.*). *Second*, the 5,623 proteins were further divided into two groups. One group included 1,859 proteins (titled ‘SameSP’) from those 17 species covered by *Training* and *Validation* datasets, and another had 3,764 proteins (titled ‘DiffSP’) from the remaining 997 species unique in ‘*Independent Testing*’ dataset. *Third*, the performances of *AnnoPRO* and those two *state-of-the-art* methods (*DeepGOPlus and PFmulDL*; *NetGO3* was not included here since its source code for model construction was not provided) were evaluated based on the two groups of ‘*Independent Testing*’ data, and the evaluating results were provided in Table 2.

**Table 2:** A comparison among those performances of *AnnoPRO* and two *state-of-the-art methods (DeepGOPlus* and *PFmulDL*) on predicting two groups of *‘Independent Testing’ data (SameSP and DiffSP)*

	**Method**	**BP**		**CC**		**MF**	
		**F**_***max***_	**AUPRC**	**F**_***max***_	**AUPRC**	**F**_***max***_	**AUPRC**
**SameSP**	*DeepGOPlus*	**0.612**	**0.593**	0.539	0.470	0.668	0.698
	*PFmulDL*	0.347	0.286	0.573	0.603	0.436	0.402
	*AnnoPRO*	0.610	0.589	**0.759**	**0.772**	**0.835**	**0.829**
**DiffSP**	*DeepGOPlus*	0.538	0.469	0.684	0.622	0.517	0.429
	*PFmulDL*	0.261	0.176	0.593	0.580	0.354	0.273
	*AnnoPRO*	**0.602**	**0.552**	**0.742**	**0.741**	**0.749**	**0.739**

*SameSP had 1,859 proteins from 17 species covered by ‘Training’ and ‘Validation’ datasets of this study; DiffSP included 3,764 proteins from the remaining 997 species unique in ‘Independent Testing’ data of this study.* Those values indicating the best performance among all three methods were highlighted in BOLD, and *AnnoPRO* performed the best in the vast majority of the Gene Ontology (GO) classes (BP, CC, MF) under both evaluating criteria (F_*max*_, AUPRC). *BP* biological process, *CC* cellular component, *MF* molecular function

As shown in Table 2, the *AnnoPRO* performed the best in the vast majority of the Gene Ontology classes (BP, CC, MF) under both evaluating criteria (F_*max*_, AUPRC), and those values indicating the best performance among those three methods (*AnnoPRO*, *DeepGOPlus*, and *PFmulDL*) were highlighted in BOLD. Particularly, for the SameSP group of *independent testing data, AnnoPRO* showed superior performance in both CC and MF with significant elevations in F_*max*_ and AUPRC (elevated by 0.13 to 0.43), and *AnnoPRO* demonstrated equivalent performance in BP comparing with *DeepGOPlus* with slightly lower F_*max*_ and AUPRC (lower by 0.002 and 0.004, respectively); for the DiffSP group of *data, the performances of AnnoPRO* stayed the best in CC and MF with significant elevation in F_*max*_ and AUPRC (elevated by 0.06 to 0.47), and the *AnnoPRO* performed better in the BP comparing with *DeepGOPlus* (F_*max*_ and AUPRC were elevated by 0.06 and 0.08). All in all, the results indicated that *AnnoPRO* gave good predictive performances on *independent* data whose species origins were covered by *training*-*validation*, and its predictive performances on *independent* data whose species origins were distinct from that of *training*-*validation*, became even better when comparing with *state-of-the-art* methods. In other words, the *AnnoPRO* showed good capacity on predicting the proteins that have little representativeness in *training*-*validation* data, which was very valuable for the function annotation of novel proteins from the species not covered by both *‘Training’ and ‘Validation’ datasets during model construction*.

### Functional annotation of the homologous proteins with distinct functions

As reported, a small variation in sequence could lead to vastly different functional outcomes [49], which made the annotation of homologous proteins with distinct functions a great challenge and a fascinating direction for the researchers in related research community. In order to evaluate the predictive performances of *AnnoPRO* and three *state-of-the-art methods* on such kind of proteins, two pairs of homologous proteins of distinct functions were then analyzed: *growth differentiation factors* (GDF8 and GDF11) and *heat shock proteins* (HSPA1A and HSPA2).

#### Case study 1 on different growth differentiation factors

**Fig. 5:**
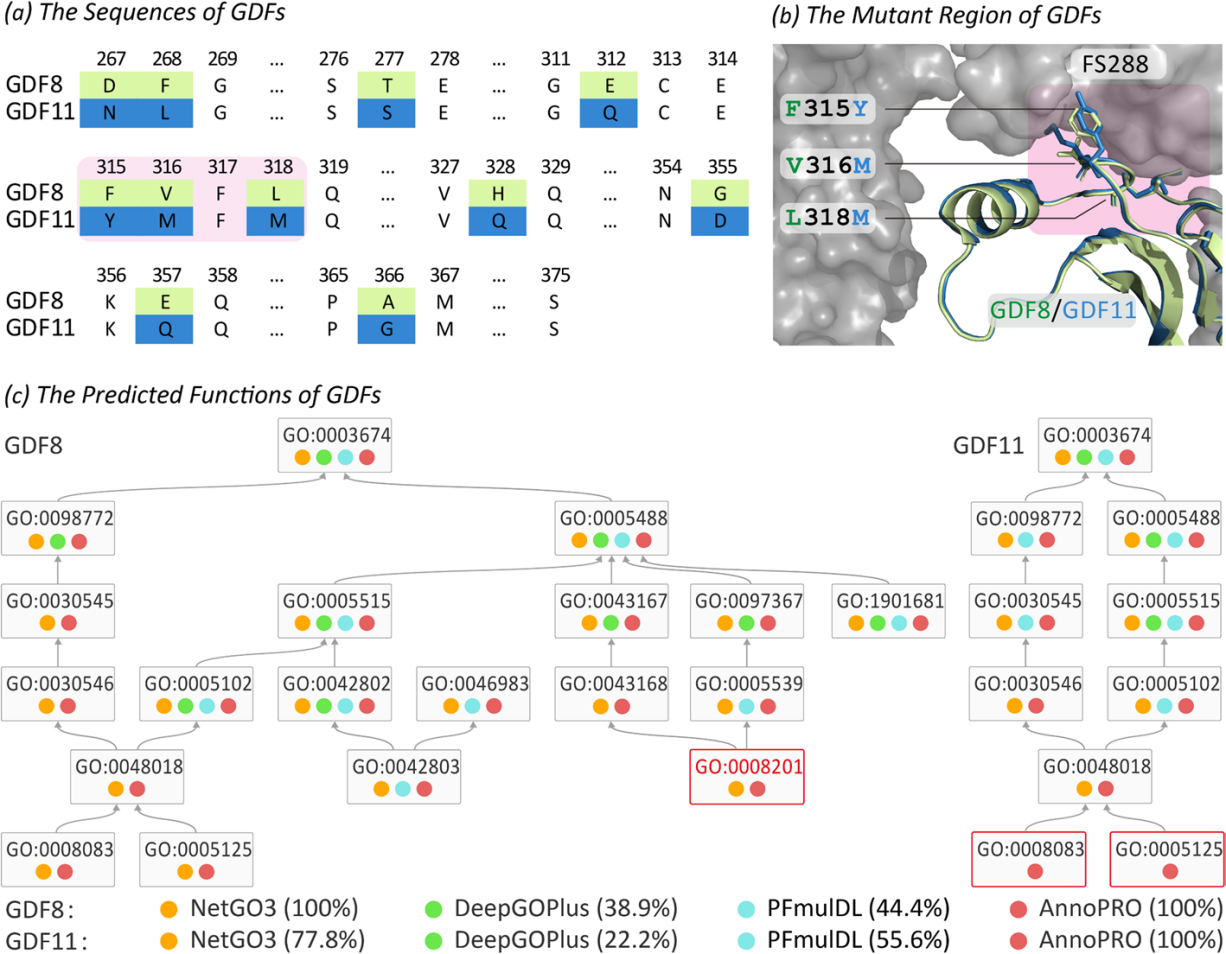
Performance assessment of four methods using two well-known *growth differentiation factors* (GDF8, GDF11). As reported, the interaction between GDF8 and follistatin-288 (FS288) formed a protein complex to bind ‘heparin’, which defined the molecular mechanisms underlying GDF8’s key GO family: ‘*heparin binding*’ (GO:0008201) [52]. Different from GDF8, the varied residues in GDF11 made it unable to interact with FS288, and it therefore suffered from the loss of the ‘*heparin binding*’ function [53]. (**a**) Sequence alignment between GDF8 and GDF11, where varied residues between two GDFs were marked in light green and blue background, respectively. Three residue pairs (F315Y, V316M, and L318M on the binding surface between the GDF8 and FS288) which were found as key residue indicating GDFs’ ‘*heparin binding*’ function [55], were given in pink background. (**b**) Structure superimposition between GDF8 (light green) and GDF11 (blue) and their interactions with FS288 (gray surface). As highlighted in pink background, three residue pairs (F315Y, V316M, L318M) located in the binding interface between GDF and FS288. (***c***) function annotation results predicted by the methods. If a GO family is successfully predicted by a method, a colored circle would be adopted to indicate that prediction result. Particularly, a successful prediction made by *AnnoPRO*, *NetGO3*, *PFmulDL* or *DeepGOPlus* was indicated by a circle of light red, orange, light blue or light green, respectively. As described, *AnnoPRO* is the only one that can successfully predict all GO families for both GDFs

*Growth differentiation factors* (GDFs) belong to the transforming growth factor β (TGFβ) family, which regulate the aspects of central nervous system (CNS) formation [50]. GDF11 (UniProt ID: GDF11_HUMAN, and UniProt accession: O95390) is a protein in the GDF family, which shares over 60% sequence similarity with GDF8 (*myostatin*, MSTN, UniProt ID: GDF8_HUMAN, and UniProt accession: O14793) and more than 90% sequence identity in the active domain [51]. As well-known, the interaction between GDF8 and *follistatin-288* (FS288) formed complex to bind heparin, which defined the molecular mechanisms underlying GDF8’s key GO family: ‘*heparin binding*’ (GO:0008201) [52]. Different from GDF8, the varied residues in GDF11 made it unable to interact with FS288, and it therefore suffered from the loss of ‘*heparin binding*’ function [53]. The sequences between GDF8’s and GDF11’s active domains were aligned in Fig. 5a, where varied residues between two GDFs were marked in light green and blue background, respectively. Combined with the structural superimpositions (as illustrated in Fig. 5b) between GDF8 (light green) and GDF11 (blue) [54], three varied residue pairs (F315Y, V316M and L318M located in the binding surface between GDF and FS288) were found key for ‘*heparin binding*’ [55].

In this study, the ‘*heparin binding*’ function (GO:0008201) for the wild type GDF8 (*GDF8-WT*) and its two mutants (*GDF8-Mutant-1* and *GDF8-Mutant-2*) was predicted using *AnnoPRO* and three *state-of-the-art tools (DeepGOPlus*, *PFmulDL*, *NetGO3)*. *GDF8-Mutant-1* contains eight mutations (D267N, F268L, T277S, E312Q, H328Q, G355D, E357Q, A366G), which locate far away from the binding interface between GDF8 and FS288. The interaction between *GDF8-WT* and FS288 forms a complex binding with heparin, which is the molecular mechanism underlying *GDF8-WT*’s ‘*heparin binding*’ function (GO:0008201). Since all eight mutations were far away from the binding interface between GDF8 and FS288, it is expected that *heparin binding* function remains in *GDF8-Mutant-1* [55]. Meanwhile, *GDF8-Mutant-2* contains three mutations (F315Y, V316M, L318M, on the binding surface between GDF8 and FS288), which were reported as the key residues indicating GDF8’s *heparin binding* function [55]. In other words, it is expected that *GDF8-Mutant-2* loses its wild type’s ‘*heparin binding*’ function [55]. All in all, there is gain-of-function of ‘*heparin binding*’ in *GDF8-WT* and *GDF8-Mutant-1*, while there is loss-of-function in *GDF8-Mutant-2*. As described in Table 3, ‘Success’ denoted that the gain/loss-of-function is successfully predicted by method, while ‘Fail’ showed that the prediction by method is incorrect. As shown, *AnnoPRO* was the only method that “successfully” captured the significant functional variations induced by small amount of residue mutations among GDF8 proteins.

**Table 3:** The prediction of the ‘*heparin binding*’ function (GO:0008201) for the wild type GDF8 (*GDF8-WT*) and two GDF8 mutants (*GDF8-Mutant-1*, and *GDF8-Mutant-2*) using *AnnoPRO* and three representative methods

**Methods**	***GDF8-WT***^a^	***GDF8-Mutant-1***^a^	***GDF8-Mutant-2***^a^
*DeepGOPlus*	Fail	Fail	Success
*PFmulDL*	Fail	Fail	Success
*NetGO3*	Success	Success	Fail
***AnnoPRO***	Success	Success	Success

‘Success’ denotes that the gain/loss-of-function is successfully predicted by the corresponding method, while ‘Fail’ indicates that it is incorrectly predicted. As demonstrated, significant functional variations among *GDF8-WT*, *GDF8-Mutant-1*, and *GDF8-Mutant-2* can only be “successfully” captured by our newly developed *AnnoPRO*

^a^Wild type GDF8 (*GDF8-WT*) is a growth differentiation factor of 375 amino acids. There are two GDF8 mutants (*GDF8-Mutant-1* and *GDF8-Mutant-2*). *GDF8-Mutant-1* contained eight mutations (D267N, F268L, T277S, E312Q, H328Q, G355D, E357Q, and A366G) which locate far away from the binding interface between GDF8 and follistatin-288 (FS288). The interaction between *GDF8-WT* and FS288 formed a protein complex to further bind to heparin. This is the molecular mechanism underlying *GDF8-WT*’s key GO term: ‘*heparin binding*’ (GO:0008201). Because all eight mutations were far away from the binding interface between GDF8 and FS288, it is expected that the ‘*heparin binding*’ function remains in *GDF8-Mutant-1* [55]. Meanwhile, *GDF8-Mutant-2* contains three mutations (F315Y, V316M, and L318M, on the binding surface between GDF8 and FS288) which are reported as the key residues indicating protein’s ‘*heparin binding*’ function [55]. In other words, it is expected that *GDF8-Mutant-2* loses its wild type’s ‘*heparin binding*’ function [55]. All in all, there is gain-of-function of ‘*heparin binding*’ in both *GDF8-WT* and *GDF8-Mutant-1*, while there is loss-of-function in *GDF8-Mutant-2*

Moreover, the sequences of GDF8 and GDF11 were reported to be highly homologous, but their functions were distinct with 291 different GO families. Therefore, it was of great interests to test the predictive performances of *AnnoPRO* and three *state-of-the-art tools* on this issue. As shown in Table 4, *AnnoPRO* performed the best in the vast-majority (11/12) of the GO classes (BP, CC, and MF) under different evaluation criteria (both recall, and precision). Taking the GO class of MF as an example (illustrated in Fig. 5c), GDF8 and GDF11 contained 19 and 10 MF families, respectively, and the functions annotated by those four methods were highlighted. If a MF family is successfully predicted by method, a colored circle will be used to indicate the prediction result. As illustrated in Fig. 5c, the successful prediction made by *AnnoPRO*, *NetGO3*, *PFmulDL* or *DeepGOPlus* was indicated by a circle of light red, orange, light blue or light green, respectively, and *AnnoPRO* is the only one that can successfully predict all MF families for both GDFs.

**Table 4:** A comparison among the predictive performances of *AnnoPRO* and three representative methods for the functional annotations of two well-known *growth differentiation factors* (GDF8, GDF11)

Protein Name	Methods	BP		CC		MF	
		**Recall**	**Precision**	**Recall**	**Precision**	**Recall**	**Precision**
GDF8	*DeepGOPlus*	0.578	0.320	0.333	**1.000**	0.389	0.333
	*PFmulDL*	0.333	0.198	0.667	0.400	0.444	0.444
	*NetGO3*	0.351	0.806	**1.000**	0.375	**1.000**	0.783
	***AnnoPRO***	**0.898**	**0.898**	**1.000**	0.731	**1.000**	**1.000**
GDF11	*DeepGOPlus*	0.402	0.306	0.625	0.714	0.222	**1.000**
	*PFmulDL*	0.404	0.494	0.875	0.412	0.556	0.833
	*NetGO3*	0.553	0.547	0.750	0.750	0.778	**1.000**
	***AnnoPRO***	**0.621**	**0.952**	**1.000**	**0.833**	**1.000**	**1.000**

Those values indicating the best performances among all methods were highlighted in BOLD, and *AnnoPRO* performed the best in the vast-majority (11/12) of the Gene Ontology (GO) classes (BP, CC, MF) under both evaluating criteria (recall, precision). All methods were ordered based on their publication dates. *BP* Biological process, *CC* Cellular component, *MF* Molecular function, *GDF8* Growth differentiation factor 8, *GDF11* Growth differentiation factor 11

#### Case study 2 on different heat shock proteins

*Heat shock proteins* (HSPs) are ubiquitous and conserved proteins in prokaryotic and eukaryotic organisms, which are essential for maintaining cellular proteostasis [56]. Herein, two *heat shock 70kDa protein* were analyzed: HSPA1A (UniProt ID: HS71A_HUMAN, and UniProt accession: P0DMV8) and HSPA2 (UniProt ID: HS71B_HUMAN, and UniProt accession: P0DMV9). The sequence similarity between HSPA2 and HSPA1A exceeds 80% (assessed using BLAST), while the total number of different GO families between these two proteins is more than 300. Therefore, it was of great interest to assess the predictive performances of *AnnoPRO* and three *state-of-the-art tools* on this particular study. As demonstrated in Table 5, our *AnnoPRO* performed the best in the vast-majority (9/12) of the GO classes under both evaluating criteria (recall and precision). Taking the GO class of MF as an example (illustrated in Additional file 1: Fig. S4 for HSPA1A and Additional file 1: Fig. S5 for HSPA2), the HSPA1A and HSPA2 had 44 and 35 MF families, respectively, and the functions annotated by those four methods were highlighted. If a MF family is successfully predicted by method, a colored circle will be used to indicate the prediction result. As illustrated, the successful prediction made by *AnnoPRO*, *NetGO3*, *PFmulDL* or *DeepGOPlus* was indicated by a circle of light red, orange, light blue or light green, respectively, and *AnnoPRO* is the only one that reach > 90% accuracies in predicting MF families for both HSPs. Furthermore, there were 16 different MF families between HSPA1A and HSPA2 (highlighted by red frames in Additional file 1: Fig. S4 for HSPA1A and Additional file 1: Fig. S5 for HSPA2). As shown, *AnnoPRO* performed the best in most (13/16) families, while *NetGO3*, *PFmulDL*, *DeepGOPlus* successfully predicted 7, 10 and 3 families, respectively.

**Table 5:** A comparison among the predictive performances of *AnnoPRO* and three representative methods for the functional annotations of two well-known *heat shock 70kDa proteins* (HSPA1A, HSPA2)

Protein Name	Methods	BP		CC		MF	
		**Recall**	**Precision**	**Recall**	**Precision**	**Recall**	**Precision**
HSPA1A	*DeepGOPlus*	0.358	0.357	0.410	0.889	0.605	0.812
	*PFmulDL*	0.635	0.457	0.615	0.800	0.814	0.500
	*NetGO3*	0.286	**0.876**	**0.634**	0.605	0.809	0.884
	***AnnoPRO***	**0.641**	0.715	0.595	**0.962**	**0.917**	**0.936**
HSPA2	*DeepGOPlus*	0.375	0.284	0.394	**0.867**	0.765	0.765
	*PFmulDL*	0.344	0.386	0.419	0.812	0.788	0.605
	*NetGO3*	0.346	0.605	0.419	0.684	0.757	0.903
	***AnnoPRO***	**0.470**	**0.851**	**0.594**	0.670	**0.868**	**0.943**

Those values indicating the best performances among all methods were highlighted in BOLD, and *AnnoPRO* performed the best in the vast-majority (9/12) of the Gene Ontology (GO) classes (BP, CC, MF) under both evaluating criteria (recall, precision). All methods were ordered based on their publication dates. *BP* biological process, *CC* cellular component, *MF* molecular function, *HSPA1A* heat shock 70 kDa protein 1A, *HSPA2 *heat shock 70 kDa protein 2

### Validating the stability of *AnnoPRO* using additional benchmark datasets

To validate the effectiveness and stability of *AnnoPRO* model, its performance was evaluated on additional datasets and compared with the SOTA methods of *PFmulDL* and *DeepGOPlus* (since *NetGO3* did not provide its training code, it could not be retrained and evaluated for comparison). Particularly, two benchmark datasets were collected from a pioneering study [32] that explicitly evaluated many strategies of protein representation. The *first* dataset was named ‘PROBE’ in the original publication [32], which consisted of 20,421 unique human proteins of distinct sequences. Following the same criterion (using Oct 22, 2019 as a *cutoff date*) used in CAFA4 for partitioning data, all these proteins were partitioned to 18,058 proteins (*adopted as ‘Training’ and ‘Validation’ datasets for model construction*) and 2,363 proteins (adopted *as ‘Independent Testing’ data*). The *AnnoPRO*, *DeepGOPlus*, and *PFmulDL* models were then retrained using these partitioned data. As shown in Table 6, *AnnoPRO* achieved the best performances on all GO classes (BP, CC, and MF), when compared with the other two models. Particularly, the F_*max*_ and AUPRC of *AnnoPRO* were substantially higher (4.5 ~ 18.8% and 4.9 ~ 24.0%, respectively) than that of two other models, which further validated its effectiveness and stability in protein function annotation.

**Table 6:** A comparison among those performances of *AnnoPRO* and two *state-of-the-art methods (DeepGOPlus* and *PFmulDL*) on constructing annotation models based on the benchmark named ‘PROBE’ in the original study [32], which consisted of 20,421 unique human proteins of distinct sequences

Method/Tool	BP		CC		MF	
	**F**_***max***_	**AUPRC**	**F**_***max***_	**AUPRC**	**F**_***max***_	**AUPRC**
*DeepGOPlus*	0.584	0.574	0.645	0.712	0.683	0.687
*PFmulDL*	0.533	0.526	0.623	0.682	0.648	0.651
*AnnoPRO*	**0.643**	**0.664**	**0.652**	**0.717**	**0.709**	**0.709**

By following the same criterion (using Oct 22, 2019 as a cutoff date) as that used by CAFA4 for data partitioning, 18,058 proteins were adopted as ‘*Training* and *Validation*’ data for model construction and 2,363 proteins were used as ‘*Independent Testing*’ dataset. The *AnnoPRO*, *DeepGOPlus*, and *PFmulDL* models were then retrained using these partitioned data. The values indicating the best performance among three methods were highlighted in BOLD, and *AnnoPRO* performed the best in all GO classes (BP, CC, MF) under both evaluating criteria (F_*max*_, AUPRC). *BP* biological process, *CC* cellular component, *MF* molecular function

**Fig. 6:**
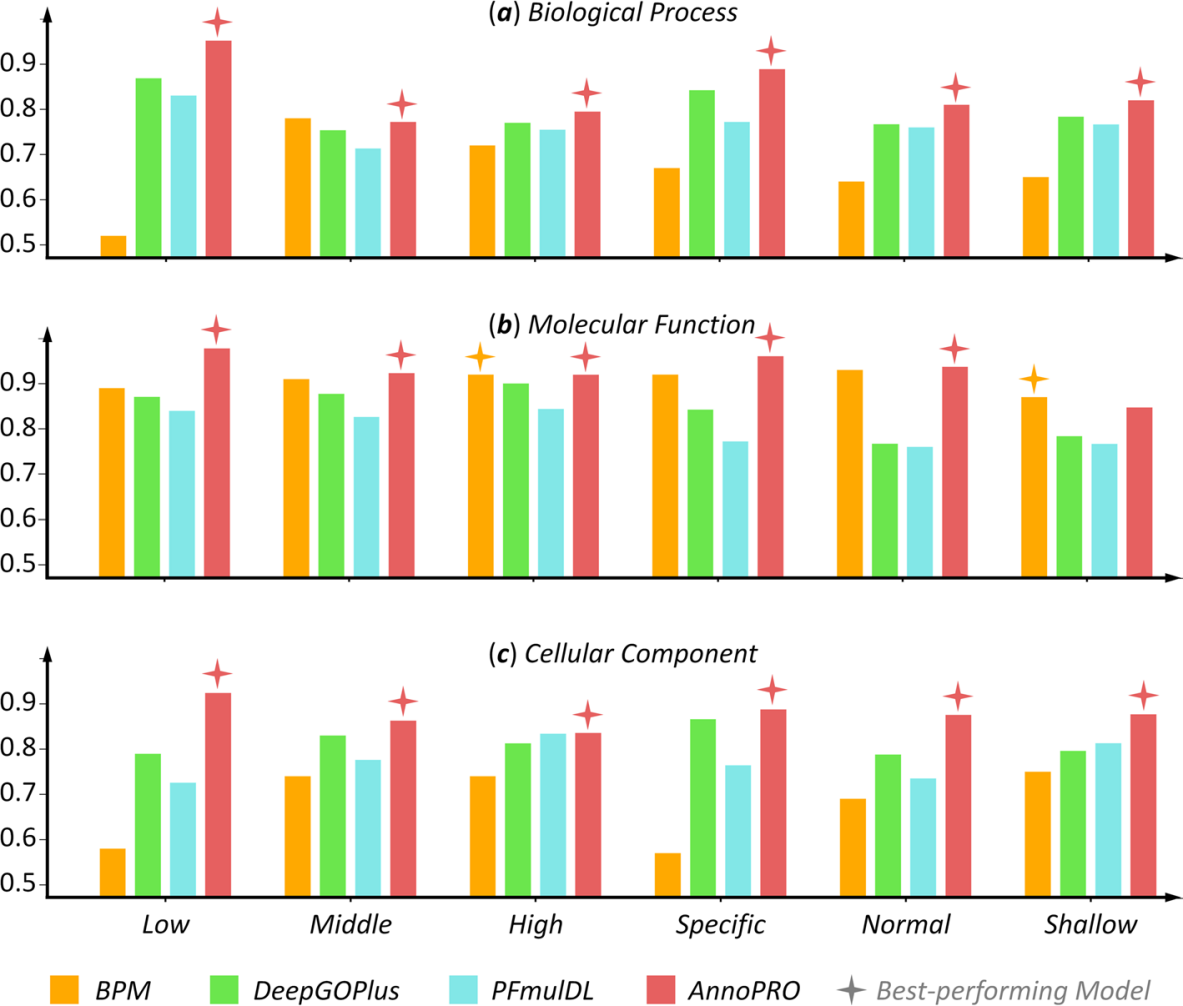
A comparison among the performances of *AnnoPRO* and three methods (*DeepGOPlus*, *PFmulDL*, and *BPM*) under six GO categories using the same sub-datasets and partition strategy as that of a previous publication [32]. *BPM*: the best-performing methods for the ‘ontology-based PFP benchmark’ in that original publication. The performances were assessed based on F_*max*_, and the performances of *AnnoPRO*, *BPM*, *DeepGOPlus*, and *PFmulDL* were highlighted in light red, orange, light green, and light blue, respectively. Each of those quadrangular-stars represented the best-performing model under a particular GO category and GO class. (**a**) *Biological Process*; (**b**) *Molecular Function*; and (**c**) *Cellular Component*. As illustrated, the *AnnoPRO* demonstrated the best performances in the vast majority (17 out of 18) of the studied GO categories

The *second* dataset was entitled ‘ontology-based PFP benchmark’ in the original publication [32], which contained 25 sub-datasets. As shown in ‘Table S5’ of that pioneering study [32], the protein representation method ‘ProtT5-XL’ gave the best-performances in most (16 out of 18) of the GO groups/categories, while the method ‘ProtALBERT’ gave the best-performances in the remaining two categories. Thus, it was of interest to compare the annotation performances among *AnnoPRO*, *DeepGOPlus*, *PFmulDL*, and the best-performing methods (BPM) under different GO categories using the same sub-datasets and partition strategy (fivefold) as that of the original publication [32]. Their performances (assessed using ‘F_*max*_’ that was the same as the original study [32]) under the 18 GO categories were provided separately in Fig. 6 according to BP, MF, and CC. As shown, *AnnoPRO* gave the best performances in most (17 out of 18) of the GO categories, which further validated the effectiveness and stability of *AnnoPRO* in functional annotation. It is necessary to emphasize that the performances of BPMs of the original publication are generated by multitask prediction model (based on SVM). If this prediction model is further optimized to the one that is well complementary to the studied protein representation method, it would be highly anticipated that the corresponding performance of functional annotation could be further elevated.

## Conclusion

Here, a novel strategy, *AnnoPRO*, was constructed by enabling *a*) the sequence-based multi-scale protein representation, *b*) the dual-path protein encoding using pre-training, and *c*) the functional annotation by LSTM-based decoding. Case studies based on benchmarks were conducted, which sufficiently confirmed the superior performance of *AnnoPRO* among available methods.

## Methods

### The collection of benchmark datasets for model construction

In this study, a total of 92,120 protein sequences were collected from the competition of CAFA4 challenge [20], and the method adopted for data partition was described in the second section of Results and Discussion. Then, the biological functions (denoted by GO families) of all proteins were matched directly from UniProt knowledgebase [4]. Like existing tools [14, 40], only those GO families with relatively large number of proteins (more than 50) were included into the model construction process of this study, which consisted of a total of 6,109 non-repetitive GO families. Moreover, the full relations among these families were downloaded from GO database [19].

Within the downloaded files, GO families were provided in a hierarchical structure. As illustrated in Additional file 1: Fig. S3, three root families were provided at the top of the structure, which included *biological process* (BP), *molecular function* (MF), and *cellular component* (CC). Then, the remaining GO families were hierarchically connected to the three root ones. In this study, the level of those root families was defined as ‘LEVEL 1’ (as shown in Additional file 1: Fig. S3). The direct child families of the root ones were classified to LEVEL 2, and the families of LEVEL 3 were determined by the direct child families of LEVEL 2. The following levels can be therefore deduced in the same manner. Based on our comprehensive evaluation on all GO data, the bottom level of GO’s hierarchical structure was LEVEL 11, which had no child family and composed of the smallest number of proteins comparing with the families in other levels (LEVEL 1 to 10). As shown in Fig. 1, the average numbers of proteins (ANP) in GO families of nine levels (LEVEL 2 to LEVEL 10) were provided. There was a clear descending trend of ANPs from LEVEL 2 to LEVEL 10. Since the ANP of one family indicated its representativeness among all families, this denoted a gradual decrease of the representativeness of a family with the penetration into deeper level. Thus, these nine levels could be classified into two groups based on their ANPs: the “*Head Label Levels*” (ANP of their GO families ≥ 2,000) and the “*Tail Label Levels*” (ANP of their GO families < 2,000). As shown, the total number (5,323) of families in “*Tail Label Levels*” was over 10 times larger than that (459) of the “*Head Label Levels*”, and such data distribution was typical for any research studies that were suffering from the ‘*long-tail problem*’ [15, 16].

### The construction of novel hybrid deep learning framework

#### Three consecutive modules integrated in the framework

As demonstrated in Fig. 2, three modules were consecutively integrated, which included: (*M1*) sequence-based module for multi-scale protein representation; (*M2*) dual-path protein encoding module based on pre-training; (*M3*) protein decoding-based functional annotation module using LSTM method. Detailed description on three modules were explicitly discussed as follows.

##### Module 1. A new sequence-based method for multi-scale protein representation

A multi-scale protein representation method was proposed to realize the conversion of sequences to *feature similarity*-based images (*ProMAP*) and *protein similarity*-based vectors (*ProSIM*). As shown in Fig. 3a, the descriptors of all CAFA4 proteins were *first* calculated using PROFEAT [34], which offered a total of 1,484 descriptors of seven classes: *amphiphilic pseudo amino acid composition*, *amino acid composition*, *molecular interaction*, *amino acid autocorrelation*, *quasi-sequence-order*, *physicochemical property* and *pseudo amino acid composition* (the descriptions on each class were shown in Supplementary Table S2). *Second*, a new *protein-descriptor matrix* (PM) was generated (provided in Fig. 3a), and any original number (${x}_{ij}^{orig}$) in this matrix was normalized to ${x}_{ij}^{norm}$ using following equation, where ${f}_{i}$ denoted the ${i}^{th}$ feature, ${\text{min}}{f}_{i}$ and ${\text{max}}{f}_{i}$ indicated the min and max value of ${i}^{th}$ feature among all proteins, respectively.

$${x}_{ij}^{norm}=\frac{{x}_{ij}^{orig}-{\text{min}}{f}_{i}}{{\text{max}}{f}_{i}-{\text{min}}{f}_{i}}$$

*Third*, the *feature distance matrix* (FDM) was produced by calculating pair-wise distances among 1,484 features using the newly generated *protein-descriptor matrix* (PM, each feature such as ${f}_{a}$ and ${f}_{b}$, was represented by a vector of 92,120 length) based on the following equation:

$$distance\left({f}_{a},{f}_{b}\right)=1-\frac{{f}_{a}\bullet {f}_{b}}{\Vert {f}_{a}\Vert \times \Vert {f}_{b}\Vert }$$

FDM was then adopted to reset the locations of protein features in a map (named ‘*template map*’), which is considered as one of the key steps in the image-like protein representation (as shown in Fig. 3a). Particularly, the process of “*feature reset*” based on FDM consisted of two key steps: ‘*dimensionality reduction*’ (by applying UMAP [36] or PCA [37] for reducing the dimensionality of each feature from 1,484D to 2D) and ‘*coordinate allocation*’ (by applying *J-V* algorithms [38] to allocate all those 1,484 features to distinct coordinates in a 39 × 39 map, named ‘*template map*’). The details on the “*feature reset*” process were further given in Supplementary Method S2.

Based on the ‘*template map*’ generated in Fig. 3a, the *ProMAP* was produced for each protein by mapping the intensities of all protein features to the corresponding locations in ‘*template map*’. As illustrated on the right side of Fig. 3b, *ProMAP* for each protein realized the transformation of ‘unordered’ vector of 1,484 protein features to the ‘ordered’ image-like representation, which is unique in capturing the intrinsic correlations among protein features and enabling a subsequent application of any deep learning methods that were popular in current image classification.

*Fourth*, a *protein distance matrix* (PDM) was further generated by calculating pair-wise distances among 92,120 proteins using the *protein-descriptor matrix* (each protein including ${p}_{a}$ and ${p}_{b}$, was represented by a vector of 1,484 length) based on the following distance equation:

$$distance\left({p}_{a},{p}_{b}\right)=1-\frac{{p}_{a}\bullet {p}_{b}}{\Vert {p}_{a}\Vert \times \Vert {p}_{b}\Vert }$$

Based on the PDM generated in Fig. 3a (highlighted in blue color), the *ProSIM* was produced for each protein by directly retrieving the corresponding column within PDM. As illustrated on the left side of Fig. 3b, the *ProSIM* of each protein realized the transformation of ‘independent’ vector of 1,484 protein features to a ‘globally-relevant’ vector of 92,120 dimensions.

##### Module 2. A novel dual-path protein encoding method based on a pre-training

In this module, a deep learning-based framework integrating *seven-channel convolutional neural network* (7C-CNN) and a *deep neural network of five fully-connected layers* (5FC-DNN) to pre-train the features of protein was adopted. Such pre-train process was expected to be effective in transferring functional family information for optimizing the concatenated protein features [57], which could extensively facilitate the application of the *long short-term memory* (LSTM) *neural network* for function annotation in next module [58]. Particularly, as illustrated in Fig. 2, the *ProMAPs* (39 × 39) for 92,120 proteins were transformed to 7 images of multi-channel based on the different classes of protein descriptor, and the multiple convolutional and max-pooling layers were used for learning the protein functions; the *ProSIMs* (92,120 × 1) for 92,120 proteins were extracted from *protein distance matrix* (PDM), and *neural network of five fully-connected layers* (5FC-DNN) was applied to encode protein sequences. By concatenating those two vectors from *ProMAP* and *ProSIM*, a total of 92,120 concatenated protein encoding vectors were created, and a fully-connected layer was further applied to refine the protein encoding by comparing with the 6,109 GO function families well-defined in *Gene Ontology*. As a result, 92,120 protein encodings were pre-trained, which were then fed into LSTM for multilabel functional annotation [33].

##### Module 3. Protein decoding-based functional annotation using LSTM method

In this module, the *long short-term memory neural network* (LSTM) was used to decode proteins for annotating their functions. LSTM had been utilized to cope with “*long-tail problem*” in *multi-label image classification* studies, since it could learn dependency among various labels [59–61]. As shown in Fig. 2, a three-layer LSTM was *first* proposed to learn hierarchical relationships among 6,109 GO families using those protein encodings pre-trained in *Module 2*. The arrows in LSTM (between any two neuros, as illustrated in Fig. 2) denoted that the value of the previous neuron (the starting point of an arrow) was adopted to adjust that of the subsequent one (the end-point of that arrow). *Finally*, ensemble learning was applied to integrate sequence similarity into functional prediction, and all proteins could be annotated into a total of 6,109 families.

### A variety of model parameters and their optimization

Various deep learning strategies were integrated into the development of *AnnoPRO* in this study, which included the *convolutional neural network* (CNN), *deep neural network* (DNN), and *long short-term memory* (LSTM). *First*, CNN contained two *convolution* layers (with their kernel size set to 3 × 3 and stride set to 1) and another two *max-pooling* layers (with their pool length set to 2 and stride set to 1). *Second*, the number of *fully-connected layers* (FC) for developing the DNN models of this study was set to 5. *Third*, the number of layers for constructing the LSTM models of this study was set to 3, and a total of 256 neurons were given for each layer. *Finally*, the input data were optimized to a time step of 11 (as shown in Additional file 1: Fig. S6). All in all, the parameters above were optimized using empirical analysis based on model performances.

During model development, a variety of parameters were optimized and systematically provided in Supplementary Table S3. *First*, 80% of 92,120 proteins from the CAFA4 benchmark dataset were selected as the training dataset, and the remaining 20% proteins were used as the validation data, which was in accordance with previous study [62]. *Then*, the ‘*mini batch size*’ and ‘*learning rate*’ for *Module 2* in Fig. 2 were given to 32 and 0.0002, respectively, with activation function for CNN and FC set to *Rectified Linear Unit* (ReLU). *Third*, ‘*mini batch size*’ and ‘*learning rate*’ of *Module 3* in Fig. 2 were also set to 32 and 0.0002, respectively, with the activation function for LSTM set to *Hyperbolic Tangent function* (Tanh) [63]. At the *end* of each training epoch, the models’ convergences on validation dataset were carefully monitored, and the model of the best performance was identified based on *early stopping* [64]. *Finally*, the *focal loss* was implemented into training process to control the direction of model optimization [65].

#### The measurements facilitating performance evaluation

Two well-established measures were adopted in this study for evaluating the model performances, which were widely adopted in the *critical assessment of functional annotation* (CAFA) challenge [20]. The measures included: *area under the precision-recall curve* (AUPRC) and *protein centric maximum F-measure* (F_max_). AUPRC is frequently applied for the evaluation of binary classifiers, especially for assessing the classes of unbalanced data, which is a numeric value between 0 and 1 [66]. The closer AUPRC is to 1, the better the prediction performance is [66]. F_max_’s strength lies in its interpretability, which is also a numeric value between 0 and 1 [20]. The closer the F_max_ is to 1, the better the prediction performance is. These two measures (AUPRC and F_max_) provided an overall performance assessment of protein functional prediction among different methods, but they were not intuitively enough for predicting a specific protein [67]. Thus, additional measures were adopted into this analysis, which included ‘*recall*’ and ‘*precision*’ [68]. Particularly, ‘*recall*’ evaluated at what percentage the true functions of a protein were successfully predicted, and the closer the recall is to 100%, the more the actual protein functions are annotated. Precision showed what percentage the predicted functions of a protein were true, and the closer the precision is to 100%, the more accurately the protein functional annotations are annotated. 

### Supplementary Information

**Additional file 1: Fig S1.** Result of Ablation experiment. **Fig S2.** Comparison among the performances of *AnnoPRO* using different dimensionality reduction methods (PCA and UMAP). **Fig S3.** Schematic illustration of the hierarchical multi-label structure of GO families (labeled by fi). **Fig S4.** Performance assessment of four methods using *Heat shock 70 kDa protein 1A* (HSPA1A). **Fig S5.** Performance assessment of four methods using *Heat shock 70 kDa protein 2* (HSPA2). **Fig S6.** A comparison of model performance using different hyperparameters. **Table S1.** AUC of nine degrees from level 1 to 9 to evaluate *AnnoPRO* and three representative methods (*DeepGOPlus*, *NetGO3* and *PFmulDL*). **Table S2.** Seven classes of protein descriptors generated using PROFEAT covered by *AnnoPRO*. **Table S3.** The hyperparameters considered in this study. **Method S1.** The Processes of Existing Methods for Model Construction. **Method S2.** The Process of Feature Reset and Its Detailed Methodology.

**Additional file 2. **Review history.

## Data Availability

The source codes for protein functional annotation using *AnnoPRO* are now available on GitHub (https://github.com/idrblab/AnnoPRO) [69] under the MIT license. It is also been deposited to Zenodo (https://zenodo.org/records/10012272) with assigned DOI: 10.5281/zenodo.10208537 [70] under the MIT license. The web-server realizing *AnnoPRO* prediction (https://idrblab.org/annopro/) was made accessible by all users, and a pypi package (https://pypi.org/project/annopro/0.1rc2/) was also provided. For the model training and testing, the available datasets of CAFA4 could be downloaded from the website (http://annopro.idrblab.cn/download/). To validate the stability of *AnnoPRO,* two benchmark datasets were obtained from a pioneering study  [71].
