# Supplementary material for: AnnoPRO: a strategy for protein function annotation based on multi-scale protein representation and a hybrid deep learning of dual-path encoding
Source: Genome Biol. 2024 Feb 1;25:41. doi: 10.1186/s13059-024-03166-1 (PMC10832132; doi:10.1186/s13059-024-03166-1)
Supplement: Supplementary file 1 — Additional file 1: Fig S1. Result of Ablation experiment. Fig S2. Comparison among the performances of AnnoPRO using different dimensionality reduction methods (PCA and UMAP). Fig S3. Schematic illustration of the hierarchical multi-label structure of GO families (labeled by fi). Fig S4. Performance assessment of four methods using Heat shock 70 kDa protein 1A (HSPA1A). Fig S5. Performance assessment of four methods using Heat shock 70 kDa protein 2 (HSPA2). Fig S6. A comparison of model performance using different hyperparameters. Table S1. AUC of nine degrees from level 1 to 9 to evaluate AnnoPRO and three representative methods (DeepGOPlus, NetGO3 and PFmulDL). Table S2. Seven classes of protein descriptors generated using PROFEAT covered by AnnoPRO. Table S3. The hyperparameters considered in this study. Method S1. The Processes of Existing Methods for Model Construction. Method S2. The Process of Feature Reset and Its Detailed Methodology. [file 13059_2024_3166_MOESM1_ESM.pdf]

*Supplementary Information for:*

# AnnoPRO: a strategy for protein function annotation based on multi-scale protein representation and a hybrid deep learning of dual-path encoding

Lingyan Zheng[1,2,†], Shuiyang Shi[1,†], Mingkun Lu[1,†], Pan Fang[2,3,†], Ziqi Pan[1], Hongning Zhang[1], Zhimeng Zhou[1], Hanyu Zhang[1], Minjie Mou[1], Shijie Huang[1], Lin Tao[4], Weiqi Xia[5], Honglin Li[6], Zhenyu Zeng[2,3], Shun Zhang[2,3], Yuzong Chen[7], Zhaorong Li[2,3,*], Feng Zhu[1,2,3,*]

[1] College of Pharmaceutical Sciences, The Second Affiliated Hospital, Zhejiang University School of Medicine, Zhejiang University, Hangzhou, 310058, China

[2] Industry Solutions Research and Development, Alibaba Cloud Computing, Hangzhou, 330110, China

[3] Innovation Institute for Artificial Intelligence in Medicine of Zhejiang University, Alibaba-Zhejiang University Joint Research Center of Future Digital Healthcare, Hangzhou, 330110, China

[4] Key Laboratory of Elemene Class Anti-Cancer Chinese Medicines, Engineering Laboratory of Development and Application of Traditional Chinese Medicines, Collaborative Innovation Center of Traditional Chinese Medicines of Zhejiang Province, School of Pharmacy, Hangzhou Normal University, Hangzhou 311121, China

[5] Pharmaceutical Department, Zhejiang Provincial People's Hospital, Hangzhou, 310014, China

[6] School of Pharmacy, East China University of Science and Technology, Shanghai, 200237, China

[7] State Key Laboratory of Chemical Oncogenomics, Key Laboratory of Chemical Biology, The Graduate School at Shenzhen, Tsinghua University, Shenzhen, 518055, China

*To whom correspondence should be addressed. Prof. Feng Zhu (zhufeng@zju.edu.cn); Mr. Zhaorong Li (zhaorong.lzr@alibaba-inc.com)

† These authors contributed equally to this work as co-first authors.

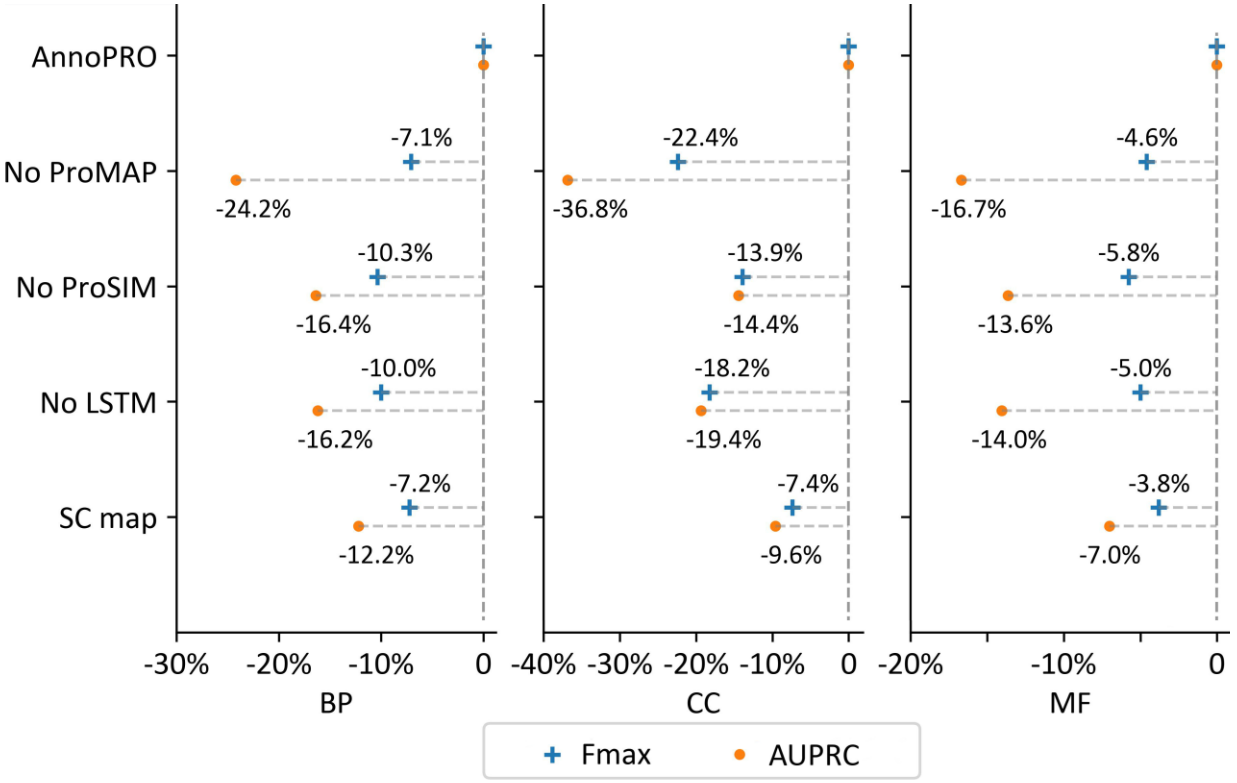


**Fig S1**. Result of Ablation experiment. The performances were represented using delta ratio of evaluating criteria ($F_{max}$ and AUPRC) in predicting the experimentally validated new protein functions that were not included in CAFA4 data, and the performances of $F_{max}$ and AUPRC were highlighted in bule plus sign and orange circle, respectively. Six comparison models were constructed and evaluated: *AnnoPRO* without *ProSIM* (No *ProSIM*), *AnnoPRO* without *ProMAP* (No *ProMAP*), *AnnoPRO* without LSTM (No LSTM), *AnnoPRO with* directly inputting the 1484 unordered features of proteins into the model (No map), *AnnoPRO* whose *ProMAP* had only one channel (Single-channel map) and *AnnoPRO* whose *ProMAP* was shuffled (shuffled map). The results showed that every change in the model algorithm led to worse results in all Gene Ontology (GO) classes (BP, CC, MF), especially the removal of the LSTM (***M 3***). In other words, *AnnoPRO* is the optimal model we had built so far. BP: *biological process*; CC: *cellular component*; MF: *molecular function*; $F_{max}$: *protein centric maximum F-measure*; AUPRC: *area under the precision-recall curve*.

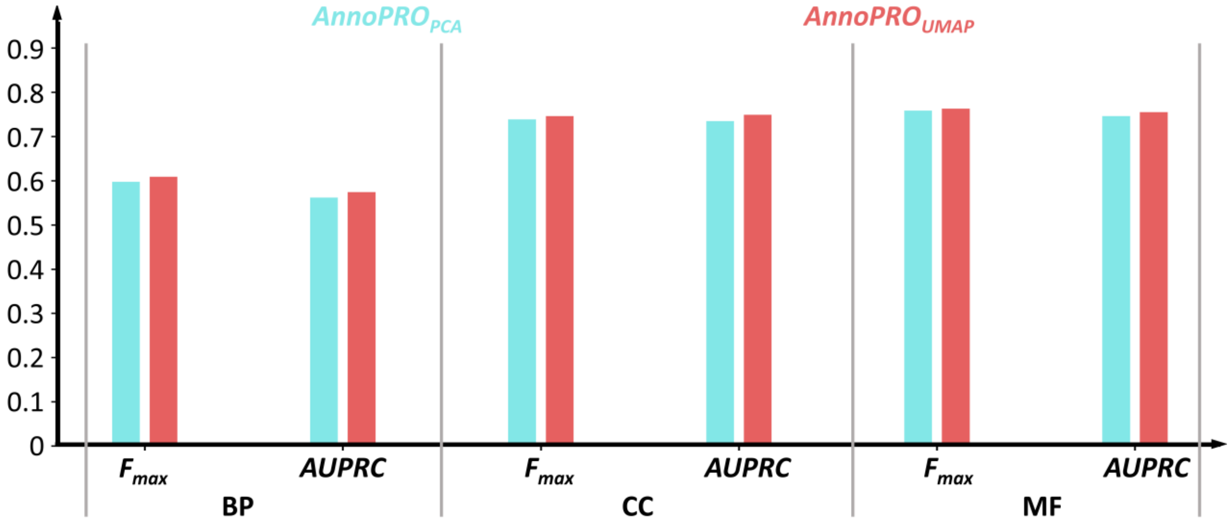


**Fig S2**. Comparison among the performances of *AnnoPRO* using different dimensionality reduction methods (PCA and UMAP). The performances were represented using $F_{max}$ and AUPRC values and the performances of *AnnoPRO$_{PCA}$*, and *AnnoPRO$_{UMAP}$* were highlighted in light blue and light red, respectively. The performances of these two models are roughly the same across three GO classes (BP, CC, MF). Particularly, *AnnoPRO$_{UMAP}$* showed a slightly better predictive performance compared with *AnnoPRO$_{PCA}$* (0.6~1.9% for Fmax; 11 1.4~2.1% for AUPRC). BP: *biological process*; CC: *cellular component*; MF: *molecular function*; $F_{max}$: *protein centric maximum F-measure*; AUPRC: *area under the precision-recall curve*.

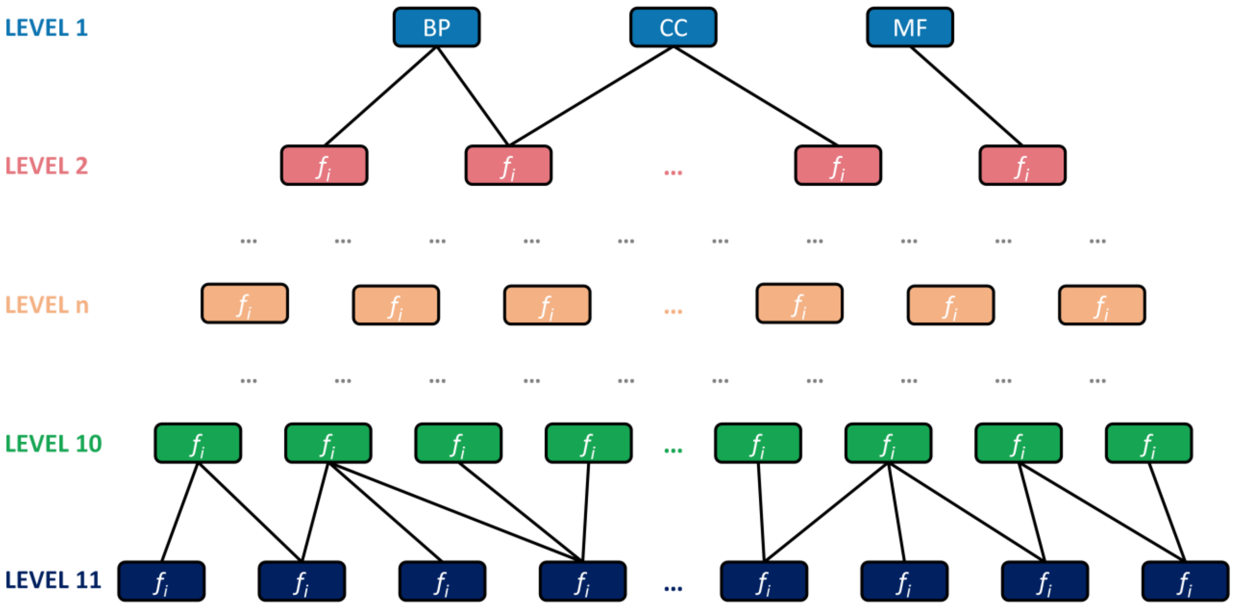


**Fig S3**. Schematic illustration of the hierarchical multi-label structure of GO families (labeled by $f_i$). Three root families were provided at the top of the structure, which included biological process (BP), molecular function (MF), and cellular component (CC), and the remaining families were hierarchically connected to them. In this study, the level of root nodes was defined as 'Level 1' (blue). The child families directly connected to the root nodes were labeled as 'Level 2' (pink). Then, the families of 'Level 3' were defined by those child families directly connected to 'Level 2'. The following levels can be thus deduced in the similar manner. Based on our comprehensive evaluation on all GO data, the bottom level of GO's hierarchical multi-label structure was 'Level 11'(blue), which had no child family and composed of the smallest number of proteins comparing with the families in other levels (Level 1 to Level 10).

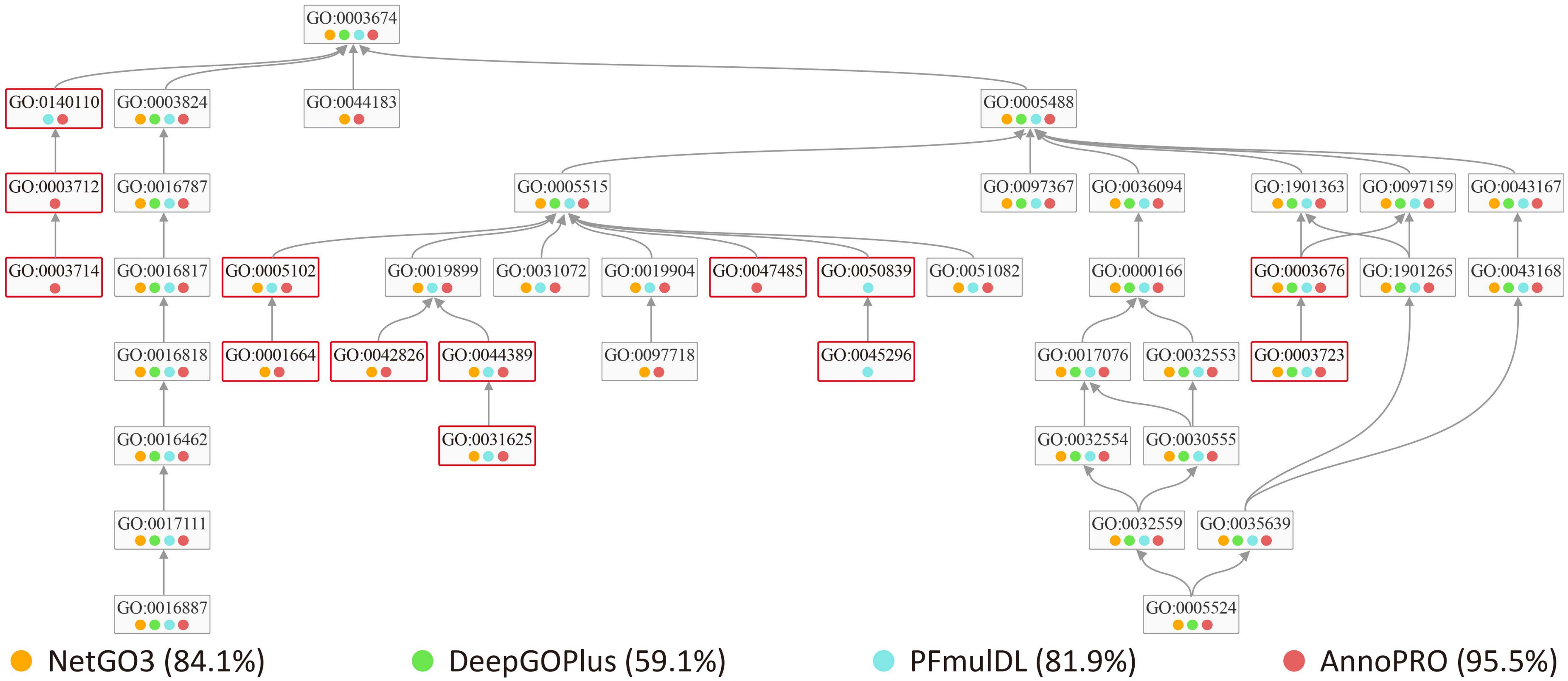


**Fig S4**. Performance assessment of four methods using *Heat shock 70 kDa protein 1A* (HSPA1A)**.** The results of functional annotation predicted by four studied methods. If a GO family is successfully prediction by one method, a colored circle will be used to indicate the prediction result. Particularly, a successful prediction made by *AnnoPRO*, *NetGO3*, *PFmulDL* or *DeepGOPlus* was indicated by a circle of light red, orange, light blue or light green, respectively. Compared with HSPA2 , another heat shock protein (represented in Fig. S7), the unique GO annotation is identified by red block. As shown, *AnnoPRO* can successfully predict most GO families for HSPA1A.

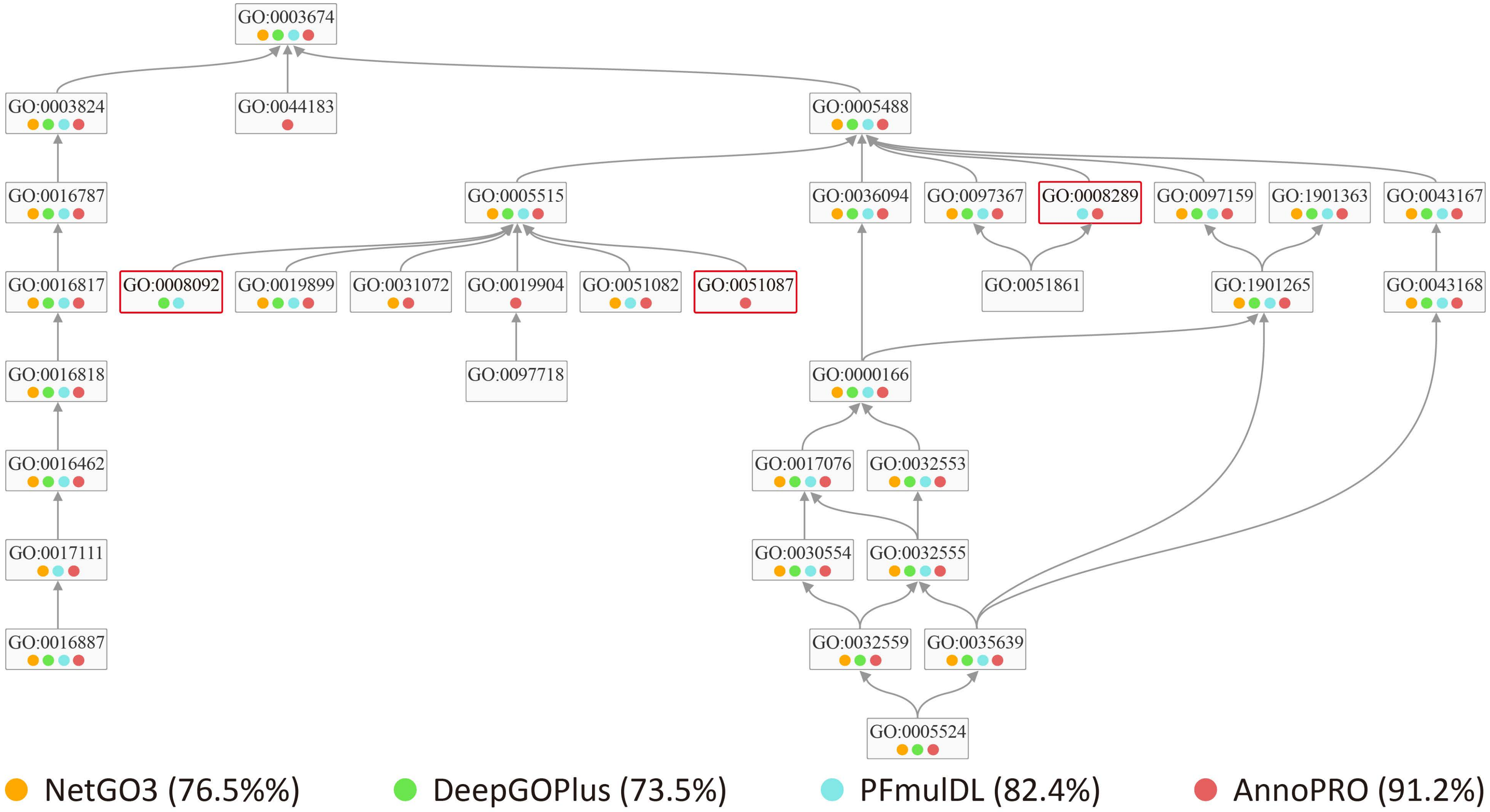


**Fig S5**. Performance assessment of four methods using *Heat shock 70 kDa protein 2* (HSPA2)**.** The results of functional annotation predicted by four studied methods. If a GO family is successfully prediction by one method, a colored circle will be used to indicate the prediction result. Particularly, a successful prediction made by *AnnoPRO*, *NetGO3*, *PFmulDL* or *DeepGOPlus* was indicated by a circle of light red, orange, light

blue or light green, respectively. Compared with HSPA1A , another heat shock protein (represented in Fig. S6), the unique GO annotation is identified by red block. As shown, *AnnoPRO* can successfully predict most GO families for HSPA2.

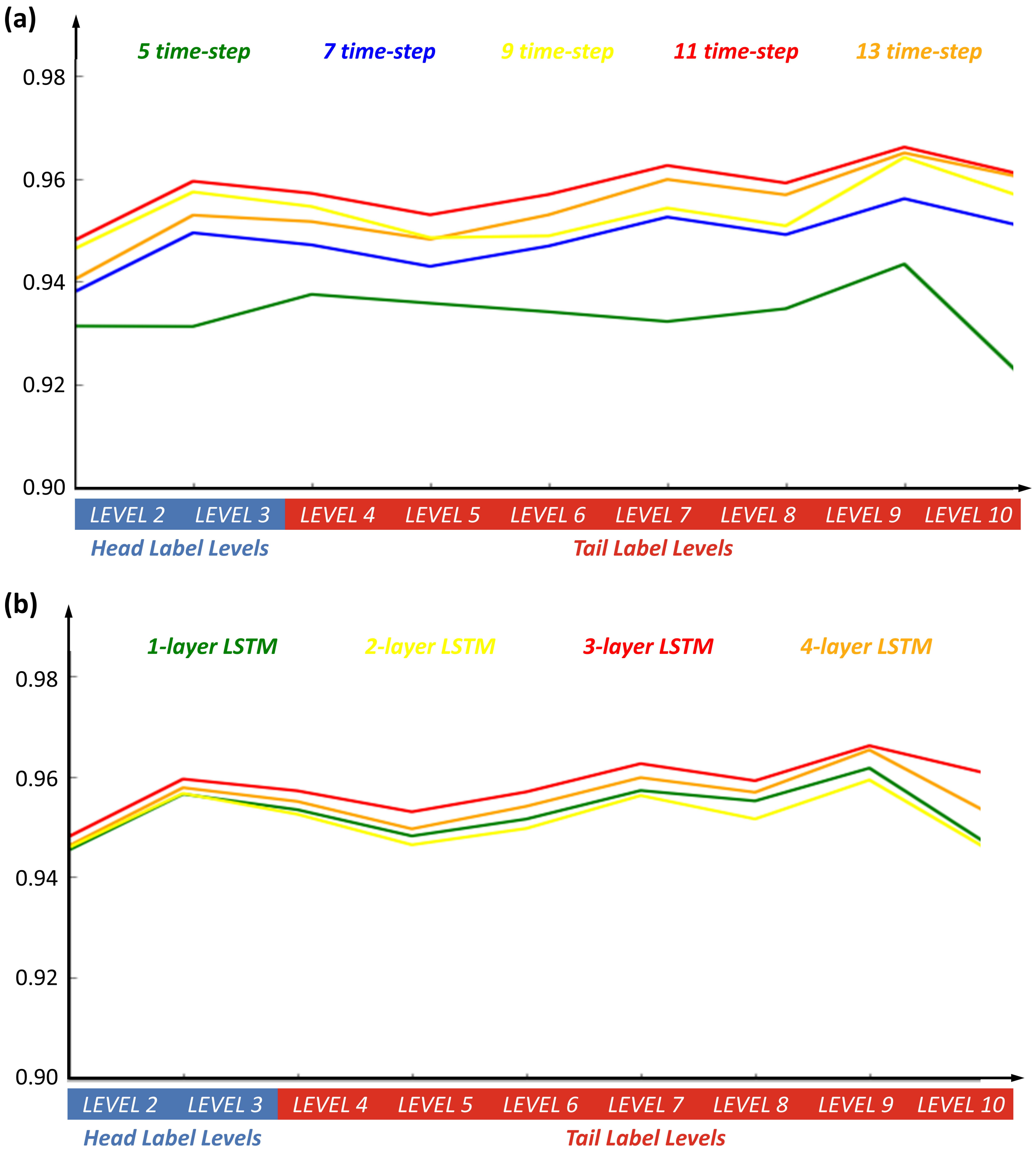


**Fig S6**. A comparison of model performance using different hyperparameters. (***a***) The graph demonstrates the effect of varying the time step on protein function prediction for the model. The performances were represented using AUC values in predicting the experimentally validated new protein functions that were not included in CAFA4 data, and performances of *AnnoPRO* with 5-, 7-, 9-, 11-, and 13-time-step input data are highlighted in green, blue, yellow, red, and orange, respectively. It is evident that *AnnoPRO* achieves the best prediction performance when using 13-time-step input data. (***b***) The figure also displays the impact of different numbers of

LSTM layers on protein function prediction for *AnnoPRO*. The performances of *AnnoPRO* with 1-, 2-, 3-, and 4-layer LSTM are highlighted in green, yellow, red, and orange, respectively. It can be observed that *AnnoPRO* achieves the highest prediction performance when using a 3-layer LSTM configuration. These results highlight the influence of hyperparameter choices on the performance of the *AnnoPRO* model. The results suggest that using 13-time-step input data and a 3-layer LSTM yield the best performance for protein function annotation.

**Table S1**. AUC of nine degrees from level 1 to 9 to evaluate *AnnoPRO* and three representative methods (*DeepGOPlus*, *NetGO3* and *PFmulDL*). Those values indicating the best performances among all methods were highlighted in BOLD, and *AnnoPRO* performed the best in the vast-majority (8/9) of the Gene Ontology classes (BP, CC, MF) under AUC. *AnnoPRO* was identified *superior* in significantly improving the annotation performances of the families in '*Tail Label Levels*' without sacrificing that of the '*Head Label Levels*', which was highly expected to make contribution to solving the long-standing '*long-tail problem*' in functional annotation.

| **Level** | **AUC** | | | |
|---|---|---|---|---|
| | ***AnnoPRO*** | ***NetGO3*** | ***PFmulDL*** | ***DeepGOPlus*** |
| Level 2 | 0.950 | **0.951** | 0.921 | 0.909 |
| Level 3 | **0.960** | 0.946 | 0.942 | 0.928 |
| Level 4 | **0.957** | 0.924 | 0.940 | 0.918 |
| Level 5 | **0.952** | 0.883 | 0.932 | 0.901 |
| Level 6 | **0.956** | 0.843 | 0.926 | 0.887 |
| Level 7 | **0.961** | 0.806 | 0.928 | 0.889 |
| Level 8 | **0.962** | 0.777 | 0.917 | 0.864 |
| Level 9 | **0.971** | 0.762 | 0.924 | 0.877 |
| Level 10 | **0.975** | 0.693 | 0.911 | 0.818 |

**Table S2**. Seven classes of protein descriptors generated using PROFEAT covered by *AnnoPRO*. These classes of descriptors could be further divided to a variety of descriptor types, and a total of 1,484 descriptors could therefore be finally generated in this study. The total number of the protein descriptors under each descriptor type was shown. The definition of each descriptor could also be found at https://github.com/idrblab/AnnoPRO.

| Descriptor Classes | Descriptor Types under Each Class | Number of Descriptors |
|---|---|---|
| Composition | Amino Acid Composition | 20 |
| | Dipeptide Composition | 400 |
| Autocorrelation | Autocorrelation descriptors | 270 |
| Interaction | CTD according to hydrophobicity | 21 |
| | CTD according to normalized vdW volumes | 21 |
| | CTD according to polarity | 21 |
| | CTD according to polarizability | 21 |
| | CTD according to charge | 21 |
| | CTD according to Molecular weight | 21 |
| | CTD according to solubility in water | 21 |
| | CTD according to No. of hydrogen bond donor in side chain | 21 |
| | CTD according to No. of hydrogen bond acceptor in side chain | 21 |
| | CTD according to CLogP | 21 |
| | CTD according to solvent accessibility | 21 |
| | CTD according to Surface tension | 21 |
| | CTD according to Amino acid flexibility index | 21 |
| | CTD according to secondary structure | 21 |

| | | |
|---|---|---|
| Physiochemical | CTD according to Protein-protein Interface hotspot propensity-Bogan | 21 |
| | CTD according to Protein-protein Interface (PPI) propensity-Ma | 21 |
| | CTD according to Protein-DNA Interface propensity-Schneider | 21 |
| | CTD according to Protein-DNA Interface propensity-Ahmad | 21 |
| | CTD according to Protein-RNA Interface propensity-Kim | 21 |
| | CTD according to Protein-RNA Interface propensity-Ellis | 21 |
| | CTD according to Protein-RNA Interface propensity-Phipps | 21 |
| | CTD according to Protein-ligand binding site propensity-Khazanov | 21 |
| | CTD according to Protein-ligand valid binding site propen-Khazanov | 21 |
| | CTD according to propensity for Protein-ligand polar and arom-Imai | 21 |
| QSO descriptors | Quasi-sequence-order descriptors | 160 |
| PAAC for AA Index Set | PAAC for amino acid index set | 50 |
| APAAC | Amphiphilic Pseudo amino acid composition | 80 |

**Table S3**. The hyperparameters considered in this study. The name of the hyperparameters and the optimized setting applied in *AnnoPRO* were explicitly described.

| **Name of the Studied Hyperparameter** | **The Setting Applied in *AnnoPRO*** |
|---|---|
| Batch Size | *32* |
| CNN Activation | *relu* |
| Dropout Rate | *0.5* |
| FC Activation | *relu* |
| Loss | *focal loss* |
| LSTM Activation | *tanh* |
| LSTM Time-step | *11* |
| LSTM Neuron Units | *256* |
| Metrics for Feature Point Distance | *cosine* |
| Method for Feature Point Embedding | *umap* |
| Monitor for Early Stopping | loss/metrics of the validation set |
| Optimizer | *Adam(lr=2e-4)* |

**Method S1**. The Processes of Existing Methods for Model Construction

Herein, we included several state-of-the-art ML-based methods for protein function prediction, namely *DeepGO*, *DeepGOCNN*, *DeepGOPlus*, *TALE*, and *PFmulDL*. For these methods, we were able to provide the training code, allowing us to ensure transparency and reproducibility in our experiments. To conduct a fair evaluation, we followed the exact training and testing procedures as described in the **Materials and Methods**. This involved using the same datasets that were utilized in the original papers and code repositories of each method. By adhering to these standardized procedures, we aimed to maintain consistency and facilitate a direct comparison of the performance of these methods. For each of the aforementioned methods, we retrained the models from scratch using our training code, following the specific methodologies outlined in their respective papers and code repositories. This ensured that our retrained models were consistent with the original implementations, allowing us to assess their performance accurately. However, it is important to note that for the models *NetGO2* and *NetGO3*, we did not have access to the training code. Therefore, we were unable to retrain these models. Instead, we evaluated and tested the existing models provided by the original authors using the same testing dataset. Although we couldn't retrain these models ourselves, we maintained a consistent evaluation framework to fairly compare their performance with the other ML-based methods. By including these state-of-the-art ML-based methods and conducting the experiments in a standardized manner, we aimed to provide a comprehensive and robust evaluation of different approaches for protein function prediction.

**Method S2**. The Process of Feature Reset and Its Detailed Methodology

The process of "*feature reset*" based on *feature distance matrix* (FDM) consisted of two key steps: '*dimensionality reduction*' (by applying UMAP or PCA for reducing the dimensionality of each feature from 1,484D to 2D) and '*coordinate allocation*' (by applying *J-V algorithms* to allocate all those 1,484 features to distinct coordinates in a 39×39 map, named '*template map*'). *First*, based on the *feature distance matrix* (FDM) (1,484×1,484), all the protein features were projected onto a 2-dimensional space as scatter points (1,484×2). Taking one feature $f_a$ as an example, it would be originally represented as a 1,484D vector ($F_a$):

$$F_a = [d_{f_{a,1}}, d_{f_{a,2}}, \dots, d_{f_{a,b}}, \dots, d_{f_{a,1484}}]$$

where $d_{f_{a,b}}$ indicated pair-wise distance between feature $f_a$ and $f_b$. *Second*, the feature vector $F_a$ was mapped into a 2D vector ($U_a$) that is easy to understand and present, by calculating their interrelationships on the manifold surface utilizing UMAP:

$$U_a = [x_{f_a}, y_{f_a}]$$

where $x_{f_a}$ and $y_{f_a}$ denoted the coordinate values of the feature $f_a$ in a two-dimensional plane as shown in **Figure 3*a***. *Third*, in order to allocate these 2D vector of protein features ($F_a$) into the Template Map, a 39×39 map ($M$) was defined to store the allocation results of the protein features. Taking the feature $f_a$ as an example, it would be represented as a grid ($M_a$) in this Template Map:

$$M_a = [m_{f_a}, m_{f_a}]$$

where $m_{f_a}$ and $m_{f_a}$were integers from 0 to 38 indicating the coordinate of the feature $f_a$. *Finally*, the grid locations of these features were allocated by minimizing the total cost between $U_a$ and $M_a$ while using the Jonker-Volgenant (J-V) algorithm:

$$[\min_{M_a} \sum_{i=1}^{N} d_{(U_a[i], M_a[i])}]$$

As a result, the 1,484 protein features were transformed from a 'unordered' vector to an 'ordered' image-like representation.
