# Supplementary material for: AnnoPRO: a strategy for protein function annotation based on multi-scale protein representation and a hybrid deep learning of dual-path encoding
Source: Genome Biol. 2024 Feb 1;25:41. doi: 10.1186/s13059-024-03166-1 (PMC10832132; doi:10.1186/s13059-024-03166-1)
Supplement: Supplementary file 2 — Additional file 2. Review history. [file 13059_2024_3166_MOESM2_ESM.docx]

**1^st^ round**

**Reviewer 1**

A major challenge in biology is to understand the function of a protein in the cell. There are many proteins in databases for which the function is unknown, and many computational approaches have been developed to predict their function. Zheng et al. have developed AnnoPRO, a method based on image-like protein representation and deep learning. The use of image-like protein sequence and feature representation is a novel approach and the comparison with other CAFA approaches show that it performs consistently better on all GO classes, in the long tail level. The authors also assessed the AnnoPRO performance by removing some of the modules that form this method which indicates that all modules are needed for the improved performance. The authors properly describe, assess, benchmark, and provide biological examples. The manuscript is novel, well written and clear. The paper is of interest to others in the field. My main concern is about the proper use/description of terminologies and other minor points:

- Please use English in the text e.g., replace "&" for "and" in line 7 of the Main section
- Regarding "So far, the functions of only 90 thousand proteins have been successfully annotated" in line 7-8 in Main section. There are more than 90 thousand proteins annotated in databases like UniProt. It seems this number comes from the CAFA dataset, which is a subset, can authors clarify in the text?
- What are GO families? This is not described in the paper and the references provided do not refer to GO families. Are these GO terms in certain protein families? It is difficult to follow the paper if certain key concepts are not clear.
- Repetition between results and discussion and methods. Please remove duplicated information
- Indicate the modules in Figure 2.
- Figure 3 is a key figure to understand the image-like representations. Please improve the description of this representation maybe with a biological example of how this is built from sequence and features.
- The examples use gene names which are diverse between species. As the analysis is based on protein sequences, I would suggest using accession numbers which are unique, for example use UniProt accessions in addition to gene names. In UniProt, heparin binding is only annotated for GDF8 (O14793) and not for GDF11 (O95390) as predicted by AnnoPro and NetGO3

**Reviewer 2**

The manuscript entitled « AnnoPRO: an innovative strategy for protein function annotation based on image-like protein representation and multimodal deep learning » by Zheng et al. reports on a deep-learning based method for predicting protein function, expressed as Gene Ontology terms. The manuscript showcases qualitative and quantitative results on reference benchmark sets, with comparison with state-of-the-art methods. The results indicate that the method outperforms the state of the art, especially for predicting lowly populated GO families. Although he work has some merit, and the method seems to have a high potential, the manuscript is its current state presents several serious issues that should be fixed.

Major concerns:

- The bibliography seems to have serious problems. For instance, refs [15-18] are cited twice as a block to support different claims. Is this choice appropriate? In what way do refs [22-24] support the division of methods in two classes? If these citations refer to methods, they should appear again later. Ref 25 is cited as a SH-based method but it is based on deep learning. To the best of my knowledge, deep learning is part of machine learning. Ref 31 seems off topic.

- The references to the figures seem incorrect in many places. For instance « Supplementary Figure S1 »on line 3, p.6 for the description of the ablation study but it actually show the GO hierarchy levels. Then Supplementary Figure 2 should be cited in support of the sentence « it is clear to see… » lines 12-14 on p. 6. « Supplementary Figure S2 «  on line 5 of p.12 should be supported fig.1.

- There are two sentences in the introduction that give estimates of the head label levels and tail label levels and the estimates are not the same. Could the authors explain these differences? Also, it is not clear in what way the cited methods address the issue of the long tail labels, as stated on p.3, lines 21-22.

- « a template image for feature reset was then generated by Jonker-Volgenant (J-V) algorithm [62] and U-Map ». Could the authors be more explicit here in explaining what the reset operation consists in and what is the main idea of the algorithm? Looking at Figure 3b, it is very difficult to get an intuition of the relationship between the two maps before and after the feature reset.

- It seems that the main component of the architecture addressing the long-tail problem is the LSTM. This is mentioned in the Methods section but it is difficult to exactly understand the underlying rationale and again, its is difficult to relate what is written in the manuscript and what is contained in the cited references. Could the authors move this part to the introduction and improve clarity? Are the 11 neurons in the LSTM enforced to match the 11 levels in the GO hierarchy? Is the output a « flat » representation of the GO hierarchy, i.e. a 6109-dimensional vector with arbitrary order?

- I had a very strange experience with the web server. The description of the method did not match that reported in the manuscript. For instance, there was some mention of protein structure descriptors and of AlphaFold2 predictions. I queried P0DP23, human calmodulin, but the server did not execute. I queried an example case provided on the main page, and the server seemed to compute forever. I could not access the results. Moreover, it was written that I had consumed 2 out of 16 free submits, suggesting that the server is not accessible freely to all. Moreover, is the source code or the architecture available and trainable?

- The method on which the work is inspired, corresponding to ref 44 was published in 2016. Could the authors provide more elements to support the fact that it is state-of-the-art as of 2023. It is also very strange that this ref 44 is not mentioned in the Methods section.

- Figure 2 and the method should be briefly described in the Results and Discussion section before the comparison with other methods. Currently, Figure 2 is introduced only with the ablation study. Please, clearly indicate M1, M2 and M3 on Figure 2. The use of multiple colors in different parts of the architecture makes the schema difficult to understand.

- Could the authors define multimodality and describe explicitly in what way their framework is multimodal? 1D vectors and 2D matrices have already been used to represent proteins and given as combined inputs to deep neural nets. What is the novelty brought by this work in that respect?

- The description of the method lacks clarity. In particular, when describing the construction of the image using « U-Map », the authors cite a publication about the Tanimoto index. In what way is that relevant? Where is the reference for « U-Map »? Did the authors use this method: L. McInnes, J. Healy, and J. Melville. Umap: Uniform manifold approximation and projection for dimension reduction, 2020. arXiv:1802.03426? To map the features in 2D according to their similarities? If yes, then the authors should discuss the limitations of U-Map. Lior Pachter's group has recently shown that UMAP produces arbitrary shapes in 2D and distort distances, which can severely bias and mislead interpretation.

- What were the training procedure used for the state-of-the-art ML-based methods the authors compare AnnoPRO with? Are they exactly the same for all methods? If yes, this should be stated more clearly. If not, this should be discussed. Were the proteins used to showcase the results, namely growth differentiation factors (GDF8 & GDF11) and heat shock proteins (HSPA1A & HSPA2) part of the training or validation set. Could the authors provide the link to the CAFA data used for training, validation and testing? The description of training/testing procedures for AnnoPRO and for the other methods should be described in the Material and Methods section.

Minor concerns:

- There are several sentences that are almost copied-pasted in multiple places throughout the text, and English writing could be improved.

- The feature similarity matrix does not encode similarities, but distances. Maybe rename it? How are the protein similarities computed?

- In the introduction, could the authors be a bit more specific about the limitations of SH-based methods? « They are severely dependent on the homology… » is kind of expected for this type of methods and does not bring much information.

- Figure S3 and S4: red fonts ans blocks. Correct to only red blocks? (The fonts are black)

**Reviewer 3**

This study describes an approach for the automated function prediction of proteins. The approach is composed of a representation scheme and a computational method. The authors claim that the described approach is particularly efficient for the proteins that has relatively small number of functional annotations.

The results and the approach is of interest to the audience. I have a major comment and a few minor remarks.

Major comment
The authors should evaluate the performance of the approach on other datasets and compare it with those of the state-of-the-art methods. For example, the datasets indicated in the following article can be employed and the performances can be compared.
Unsal, S., Atas, H., Albayrak, M. et al. Learning functional properties of proteins with language models. Nat Mach Intell 4, 227-245 (2022). https://doi.org/10.1038/s42256-022-00457-9

Minor remarks
Pp3, lines 5-6
Please explain.
"Compared with protein sequences, the acquirement of experimentally-validated protein functions is much more challenging …"

Pp3, lines 7-8
Please cite the reference for 90,000 proteins whose functions have been successfully annotated. Can you also check if this number is up to date?
"So far, the functions of only 90 thousand proteins have been successfully annotated, …"

Pp4, line 13
Can you show how the method is capturing the intrinsic correlations among protein features?
"method is unique in capturing the intrinsic correlations among protein features"

**Authors’ response**

**Comment 1**

As you will see from the reports, all referees are broadly favorable and find the work of potential interest, but they all raise important issues that we must ask you to address, in the form of a revised manuscript, before we reach a final decision on publication. In particular, all referees request additional clarifications and explanations.

**Response to Comment 1**

Thanks a million for these encouraging comments. During the revision, we have made point-to-point response to the valuable comments of you and three reviewers, and corresponding revisions were made to both the revised manuscript (highlighted in BLUE) and the source codes.

**Comment 2**

Referee 2 raises concerns that the citations often do not match what they are being used to support, and also that there appears to be limitations to using the method.

**Response to Comment 2**

Thanks a million for pointing out these problems. ***First***, we agree with referee 2 that some of the citations are inappropriately offered in our original submission. During the revision, all citations were carefully reviewed to check whether they fully matched what they are being used to support, and the incorrect citations were replaced by appropriate ones. All in all, in the revised manuscript, a total of 68 citations were included, and they have been carefully validated that they fully match what they are being used to support.

***Second***, thanks for referee 2 to point out the limitations on using our online method, which were caused by a temporary network interruption between our web interface and core calculation unit. As suggested by referee 2, our online method (web-server) was reconstructed during the revision to upgrade its network connection, improve user-friendly interface, ensure unlimited access, and provide professional manual and introductions. We felt sorry for the inconvenience caused to our referee, and we promise to persistently maintain and upgrade the functionality of our online web-server, which is now freely accessible to all users at: <https://idrblab.org/annopro/>

**Comment 3**

Note the Referee 3 has asked that you benchmark against additional datasets. We agree that this would strengthen the manuscript, and we would be happy for you to include this extra work, but based on the reports from the other reviewers, we would not make it a requirement.

**Response to Comment 3**

We would like to thank the Reviewer 3 for these constructive comments. During the revision, a literature review on that suggested publication (*Nature Mach Intell*. 4: 227, 2022) was conducted, and two benchmarks were collected from that pioneering study to further assess the effectiveness and stability of *AnnoPRO*. The ***first*** benchmark was named PROBE in the original study (*Nature Mach Intell*. 4: 227, 2022), which contained 20,421 unique human proteins of distinct sequences. Following the same criterion (using Oct 22, 2019 as a *cutoff date*) used in CAFA4 for partitioning data, all these proteins were partitioned to 18,058 proteins (adopted as ‘*Training*’ and ‘*Validation*’ datasets for model construction) and 2,363 proteins (adopted as *Independent Testing*). *AnnoPRO* and the SOTA methods (such as *DeepGOPlus* and *PFmulDL*; Since *NetGO3* did not provide its training codes, it could not be retrained and evaluated for comparison here) were then completely retrained using those partitioned data described above. As shown in **Table 6**, *AnnoPRO* achieved the best performance on all GO classes, when compared with other two models. Particularly, the F*_max_* and AUPRC of *AnnoPRO* were much higher (+4.5~+18.8% and +4.9~+24.0%, respectively) than that of two other models, which validated its effectiveness in function annotation.

The ***second*** dataset was entitled ‘ontology-based PFP benchmark’ in that pioneering publication (*Nature Mach Intell*. 4: 227, 2022), which consisted of 25 sub-datasets. As described in the ‘*table S5*’ of that study, a protein representation method named ‘ProtT5-XL’ gave the best-performance in most (16 out of 18) of the GO groups/categories, while the method called ‘ProtALBERT’ gave the best-performance in the remaining categories. Thus, it is of interest to compare the annotation performances among *AnnoPRO*, *DeepGOPlus*, *PFmulDL*, and best-performing methods (BPMs) under the 18 categories using the same sub-datasets and partition strategy (5-fold cross validation) as that of the original study. Their performances (assessed using F*_max_*, which was the same as the original study) under those 18 GO categories were provided separately in **Figure 6** based on BP, MF, and CC. As demonstrated, *AnnoPRO* gave the best performances in most (17 out of 18) GO categories, which further showed the effectiveness and stability of *AnnoPRO* in protein function annotation. It is necessary to emphasize that the performances of BPMs of the original study are generated by an SVM-based prediction model. If this model can be further optimized to the one that is well complementary to the studied protein representation method, it would be anticipated that the corresponding performance of functional annotation could be further elevated.

All in all, we would like to express our great appreciation to Reviewer 3 for the very constructive suggestion. Although it took plenty of GPU computing time, the revision based on these valuable comments further validated the effectiveness and stability of *AnnoPRO* on additional benchmark, and compared *AnnoPRO* with those of the *state-of-the-art* methods. Modifications were made at the end of “**Results and Discussion**” by adding two new paragraphs (highlighted in BLUE), and one new table (**Table 6**) and another new figure (**Figure 6**) were added to the main text.

**Comment 4**

We are considering your manuscript as a 'Method' article. This is our format for publishing articles that describe a methodological innovation that is a significant advance over published methods and likely to be of broad utility, but that do not provide significant biological insights. When revising the manuscript, please ensure the manuscript conforms to our style for Methods articles; specifically, the abstract should be under 100 words. Please note that if we decide to publish your manuscript, we will require that the source code is made publicly available under an open-source license compliant with Open-Source Initiative, with the license clearly stated in the manuscript. The source code should be deposited in a public repository, such as for instance GitHub, with the accession links included in the manuscript. We also ask that the version of source code used in the manuscript is deposited in a DOI-assigning repository, such as Zenodo, with the link also included. All this information should be listed in a separate Availability of Data and Materials section of the manuscript.

**Response to Comment 4**

Thanks for your valuable suggestions. We are interested in publishing our research as a ‘Method’ article. During the revision, we adhered to the writing style requirements of *Genome Biology* for Method, such as the compression of Abstract to <100 words, and the open accessibility of source codes on public repository GitHub (<http://github.com/idrblab/AnnoPRO>) and the DOI-assigning repository Zenodo (<https://zenodo.org/records/10012272>). Detailed information was provided in the section of “**Availability of Data and Materials**” in our revised manuscript.

**Responses to Reviewer 1**

**Comment 1**

A major challenge in biology is to understand the function of a protein in the cell. There are many proteins in databases for which the function is unknown, and many computational approaches have been developed to predict their function. Zheng et al. have developed AnnoPRO, a method based on image-like protein representation and deep learning. The use of image-like protein sequence and feature representation is a novel approach and the comparison with other CAFA approaches show that it performs consistently better on all GO classes, in the long tail level. The authors also assessed the AnnoPRO performance by removing some of the modules that form this method which indicates that all modules are needed for the improved performance. The authors properly describe, assess, benchmark, and provide biological examples. The manuscript is novel, well written and clear. The paper is of interest to others in the field. My main concern is about the proper use of terminologies and other minor points.

**Response to Comment 1**

Thanks a million for your encouraging comments. During the revision, we have made point-to-point responses to your valuable comments, and corresponding revisions are made to the revised manuscript (highlighted in BLUE) and the provided source codes.

**Comment 2**

- Please use English in the text e.g., replace "&" for "and" in line 7 of the Main section

**Response to Comment 2**

Thanks for pointing out the language issue in our original submission, which has been corrected in our revised manuscript. Moreover, during the revision, we carefully proof-read the entire main text of manuscript, and improved the language by consulting our colleagues. The native language of our colleagues is English, and they are from either my research directions or school of foreign languages in my university. Based on their suggestion, modifications were made throughout the manuscript and highlighted in BLUE color. Thanks a million for your valuable comments.

**Comment 3**

- Regarding "So far, the functions of only 90 thousand proteins have been successfully annotated" in line 7-8 in Main section. There are more than 90 thousand proteins annotated in databases like UniProt. It seems this number comes from the CAFA dataset, which is a subset, can authors clarify in the text?

**Response to Comment 3**

We would like to thank the reviewer for these professional comments. In our original submission, the number 90,000 was adopted from a previous publication (*Nucleic Acids Res*. 49:W469, 2021), which greatly increased from the number of 66,000 reported by another study (*Genome Biol*. 20: 244, 2019). Such result indicated that the number of proteins of experimental function annotation kept growing over time. Therefore, during the revision, we conducted a careful literature review to check whether the number (90,000) described in our original submission was still up-to-date. As described in the latest release of UniProt (*Nucleic Acids Res*. 51: D523, 2023), about 100,000 proteins that were manually annotated based on experimental evidence, were collected to current UniProt. In other words, it is difficult to provide “up-to-date” statistics as the numbers may differ among different studies. Thus, in the revised paper, we modified the corresponding part to:

“So far, only a very small portion of protein sequences have been successfully annotated based on experimental evidence [4], …”, and citation [4] is *Nucleic Acids Res*. 51: D523, 2023.

All in all, we thank reviewer 1 for the valuable comment, which made the current version of our manuscript much more academically rigorous than our previous submission.

**Comment 4**

- What are GO families? This is not described in the paper and the references provided do not refer to GO families. Are these GO terms in certain protein families? It is difficult to follow the paper if certain key concepts are not clear.

**Response to Comment 4**

Thanks a million for your valuable comments. In this study, those “GO families” referred to “GO terms” in *Gene Ontology* (GO) database. The so-called “gene ontology families” or “GO families” were frequently reported in recent publications, such as: *Nat Commun*. 13: 6816, 2022; *Cell Rep*. 38:110381, 2022; *Commun Biol*. 4:1292, 2021; *Front Oncol*. 10: 575737, 2020; *Circulation*. 139: 2466, 2019; *Chem Res Toxicol*. 33: 1121, 2020. We felt sorry for not clarifying the key concepts in our previous submission. In the revised manuscript, the corresponding section was re-written, and modifications were made to the second paragraph of **Background** (highlighted in BLUE):

“However, the annotation of protein function using computational method has been suffering from the serious “*long-tail problem*” [14-16]. Based on the current *Gene Ontology* (GO) database [17], the average numbers of proteins (ANP) in those GO families (terms) of different GO levels were assessed and statistically described in **Figure 1**, and the number 2,000 was set as a cutoff of ANP for differentiating ‘*Tail Label Levels*’ from ‘*Head Label Levels*’”

**Comment 5**

- Repetition between results discussion and methods. Please remove duplicated information

**Response to Comment 5**

Thanks a million for reminding us to remove the duplicated description among results, discussion and methods. During the revision, a careful review on our manuscript and the corresponding text editing were conducted to provide different descriptions among these sections. All corresponding modifications were highlighted in BLUE color in our revised manuscript. We really appreciated that you brought this issue to our attention during the peer-review process, and the latest version of our manuscript became much clearer compared with our previous submission. Thanks!

**Comment 6**

- Indicate the modules in Figure 2.

**Response to Comment 6**

We would like to thank the reviewer 1 for this valuable comment. During the revision, **Figure 2** was modified by indicating each of the three modules. Furthermore, a new **Section 2.1** was added at the beginning of **Results and Discussion** for briefly describing each module in **Figure 2**. The newly added paragraph in **Section 2.1** was provided below for your consideration:

Herein, a hybrid deep learning framework was constructed to enable protein function annotations, which consisted of three consecutive modules (***M1*** to ***M3***). As shown in **Figure 2**, these modules included (***M1***) the sequence-based multi-scale protein representation realizing the conversion of all protein sequences to *feature similarity*-based images (*ProMAP*) and *protein similarity*-based vectors (*ProSIM*). Particularly, at *feature similarity* scale, the similarities among protein features were utilized to transform the ‘unordered’ vector of 1,484 protein features to an ‘ordered’ image-like representation; at *protein similarity* scale, the pair-wise similarities between sequences were adopted to transform the ‘independent’ vector of 1,484 protein features to a ‘globally-correlated’ vector of 92,120 dimensions. (***M2***) the dual-path protein encoding based on a pre-training. Using the *ProMAP* and *ProSIM* generated for all proteins, a dual-path encoding was constructed based on a seven-channel *Convolutional Neural Network* (7C-CNN) and *Deep Neural Network* of five fully-connected layers (5FC-DNN) to pre-train the features of all CAFA4 proteins by integrating their annotation data of GO families. (***M3***) the functional annotation by a LSTM-based decoding. The protein features pre-trained using the dual-path encoding layer in ***M2*** were concatenated and then fed into a *long short-term memory recurrent neural network* (LSTM) to enable a multi-label annotation of proteins to 6,109 functional GO families using the hybrid deep learning. The details of this hybrid deep learning framework were further elaborated in **Materials and Methods**.

**Comment 7**

- Figure 3 is a key figure to understand the image-like representations. Please improve the description maybe with a biological example of how this is built from sequence and features.

**Response to Comment 7**

Thanks a million for this very constructive suggestion. During the revision, **Figure 3** were further improved according to your valuable suggestion by adding descriptions and providing biological examples on how the representation is built. Moreover, a new paragraph was added at the end of **Section 2.1** to extensively describe **Figure 3** (highlighted in BLUE and provided below).

In the hybrid deep leaning framework, the sequence-based multi-scale protein representation was one of the key modules (***M1***). As shown in **Figure 3**, the way how the conversion of all sequences to *feature similarity*-based images (*ProMAP*) and *protein similarity*-based vectors (*ProSIM*) was described. On the one hand, a method realizing image-like protein representations was proposed (*ProMAP*) for capturing the intrinsic correlations among protein features. As illustrated in **Figure 3*a***, a template map for each protein sequence was *first* constructed by a consecutive process of ‘*protein representation*’ (using PROFEAT [34]), ‘*similarity calculation*’ (using *Cosine Similarity* [35]), ‘*dimensionality reduction*’ (using UMAP [36] or PCA [37]), ‘*allocation algorithms*’ (using *Jonker-Volgeant algorithm* [38]), *etc.* *Then*, *ProMAP* was produced for each protein by mapping the intensities of all protein features to their corresponding locations in the constructed template map (illustrated on the right side of **Figure 3*b***). On the other hand, an approach considering the global relevance among proteins was proposed (*ProSIM*) to convert the ‘independent’ vector to a ‘globally-relevant’ protein representation. As illustrated in **Figure 3*a***, a protein distance matrix (PDM) was *first* generated by following a consecutive process of ‘*protein representation*’ (using PROFEAT [34]) and ‘*similarity calculation*’ (using *Cosine Similarity* [35]). *Finally*, *ProSIM* was generated for each protein by retrieving directly from each row of the newly generated PDM (as shown in the left side of **Figure 3*b***). All in all, these newly proposed strategies could capture the intrinsic correlation among protein features and consider the global relevance among sequences. The detailed description was explicitly provided in the **Materials and Methods**.

**Comment 8**

- The examples use gene names which are diverse between species. As the analysis is based on protein sequences, I would suggest using accession numbers which are unique, for example use UniProt accessions in addition to gene names. In UniProt, heparin binding is only annotated for GDF8 (O14793) and not for GDF11 (O95390) as predicted by AnnoPRO and NetGO3.

**Response to Comment 8**

We would like to the thank the reviewer for the very professional comments. During the revision, both UniProt accession and UniProt ID were provided for those gene names, and corresponding modifications were made to the first paragraph of both **Section 2.5.1** and **Section 2.5.2**.

All these modifications were highlighted in BLUE color.

**Responses to Reviewer 2**

**Comment 1**

The manuscript entitled “AnnoPRO: an innovative strategy for protein function annotation based on image-like protein representation and multimodal deep learning” by Zheng et al. reports on a deep-learning based method for predicting protein function, expressed as Gene Ontology terms. The manuscript showcases qualitative and quantitative results on reference benchmark sets, with comparison with state-of-the-art methods. The results indicate that the method outperforms the state of the art, especially for predicting lowly populated GO families. Although the work has some merit, and the method seems to have a high potential, the manuscript is its current state presents several serious issues that should be fixed.

**Response to Comment 1**

Thanks a million for your valuable comments. During the revision, we have made point-to-point responses to these professional questions, and a variety of corresponding revisions were made to the revised manuscript (highlighted in BLUE) and the provided source codes.

**Comment 2**

Major: - The bibliography seems to have serious problems. For instance, refs [15-18] are cited twice as a block to support different claims. Is this choice appropriate? In what way do refs [22-24] support the division of methods in two classes? If these citations refer to methods, they should appear again later. Ref 25 is cited as SH-based method but it is based on deep learning. To the best of my knowledge, deep learning is part of machine learning. Ref 31 seems off topic.

**Response to Comment 2**

Thanks a million for pointing out the problems. During the revision, (***a***) the corresponding parts citing Refs [15-18] in previous submission were modified using more appropriate citations:

“which extensively promote the identification of efficacious drug target [12], the revealing of molecular mechanism underlying sophisticated disease etiology [13], and so on”

“However, the annotation of protein function using computational method has been suffering from the serious “*long-tail problem*” [14-16]”

(***b***) the corresponding section citing Refs [22-24] were modified using more appropriate citation:

“So far, two types of protein function annotation strategy have been constructed, which can be roughly divided into the sequence homology (SH) based ones and the machine learning (ML) based ones [21]”

It was clearly stated in Ref [21] (*Sci Rep*. 11: 1160, 2021): “Computational biology has been bridging the sequence-annotation gap for decades, based on two different concepts: (1) sequence similarity-based transfer (or homology-based inference) … (2) De-novo methods predict protein function through machine learning”

(***c***) the corresponding section citing Ref [25] were modified using more appropriate citation:

“SH-based strategy has long been used for protein function annotations [22]”

It was explicitly stated in Ref [22] (*Int J Mol Sci*. 19: 183, 2018): “Among these in silico methods, the basic local alignment search tool (BLAST) revealing protein functions based on excess sequence similarity demonstrated great capacity and attracted substantial interest from the researchers of this field”

(***d***) the corresponding section citing Ref [31] were modified using more appropriate citation:

“but the accuracy of sequence alignments drops off rapidly in cases where the sequence identity/homology falls below certain critical point [25]”

Ref [25] (*Genome Biol*. 18: 186, 2017) stated: “the accuracy of sequence alignments drops off rapidly in cases where the sequence identity falls below a certain critical point”

Moreover, during the revision, we conducted a systematic review of all 68 references in the latest version of our paper to check whether they fully matched what they are being adopted to support, and the incorrect citations were replaced by appropriate ones. All in all, in the revised manuscript, a total of 68 citations were included, and they have been carefully validated that they fully match what they are being used to support.

**Comment 3**

Major: - The references to the figures seem incorrect in many places. For instance, “Supplementary Figure S1” on line 3, p.6 for the description of the ablation study but it actually show the GO hierarchy levels. Then Supplementary Figure 2 should be cited in support of the sentence “it is clear to see…” lines 12-14 on p. 6. “Supplementary Figure S2” on line 5 of p.12 should be supported fig.1.

**Response to Comment 3**

We felt very sorry that the references to the supplementary figures were incorrect in our previous submission, and the **Supplementary Figure S1** and **Supplementary Figure S2** should exchange with each other, which was corrected in our revised paper. Moreover, during the revision, all the references to figures and tables were carefully checked. Thanks for pointing out this problem.

**Comment 4**

Major: - There are two sentences in introduction that give estimates of the head label levels and tail label levels and the estimates are not the same. Could the authors explain these differences?

**Response to Comment 4**

Thanks a million for the valuable comments. There were two sentences in the **Background** that gave estimates of the ‘*Head Label Levels*’ and ‘*Tail Label Levels*’, which are provided below.

In the 2nd paragraph: “As shown in **Figure 1**, the total number (5,323) of GO families in ‘*Tail Label Levels*’ is more than 10 times larger than that (459) of ‘*Head Label Levels*’ [17]”

In the 3rd paragraph: “However, due to the overwhelming domination of proteins in the ‘*Head Label Levels*’ (the average number of proteins in the family of *Head Label Levels* equals to 4,210, which is about 5 times larger than that (886) of *Tail Label Levels*, as shown in **Figure 1**), it is still extremely challenging for existing methods/tools to improve the prediction accuracies for the families in *Tail Label Levels*, and the “*long-tail problem*” in protein functional annotation remains unsolved [32]”

These sentences together with **Figure 1** gave estimations of the differences between ‘*Head Label Levels*’ and ‘*Tail Label Levels*’ from two different perspectives. For the first perspective, the total number of GO families in ‘*Tail Label Level*’ was compared with that of ‘*Head Label Level*’ (in the 2nd paragraph), which resulted in the estimation of “more than 10 times larger than”. For the second perspective, the average number of proteins (ANP) in each family of ‘*Head Label Levels*’ was compared with that of ‘*Tail Label Levels*’, which resulted in the estimation of “about 5 times larger than”. Since these two perspectives were very different from each other, the corresponding estimations were not the same. Again, we would like to thank you for the valuable comments.

**Comment 5**

Also, it is not clear in what way the cited methods address the issue of the long tail labels, as stated on p.3, lines 21-22.

**Response to Comment 5**

We would like to thank you for the valuable comments, and felt sorry that we did not clarify this in our previous submission. In the revised paper, the corresponding sentence was corrected to:

“So far, two types of protein function annotation strategy have been constructed, which can be roughly divided into the sequence homology (SH) based ones and the machine learning (ML) based ones [21]”

In other words, the cited method was not designed to specifically address the issue of ‘*long-tail problem*’. All corresponding modifications were made at the end of the second paragraph of the **Background** section, and all modifications were highlighted in BLUE color.

**Comment 6**

Major: - “a template image for feature reset was then generated by Jonker-Volgenant (J-V) algorithm [62] and U-Map”. Could the authors be more explicit here in explaining what the reset operation consists in and what is the main idea? Looking at Figure 3b, it is very difficult to get an intuition of the relationship between the two maps before and after the feature reset.

**Response to Comment 6**

We would like to thank reviewer 2 for giving us this opportunity to further clarify what the reset operation consists in. In this study, a multi-scale protein representation method was proposed to realize the conversion of a protein sequence to a *feature similarity*-based image (*ProMAP*). ***First***, the *feature distance matrix* (FDM) was produced by calculating pair-wise distances among 1,484 features using the *protein-descriptor matrix* (PM) shown in **Figure 3*a***. ***Then***, FDM was adopted to reset the locations of protein features in a map (named ‘*template map*’), which was considered as one of the key steps in the image-like protein representation (shown in **Figure 3*a***). Particularly, the process of “*feature reset*” based on FDM contained two key steps: ‘*dimensionality reduction*’ (by applying UMAP or PCA for reducing the dimensionality of each feature from 1,484D to 2D) and ‘*coordinate allocation*’ (by applying *J-V algorithm* to allocate those 1,484 features to distinct coordinates in a 39×39 map, named ‘*template map*’). During the revision, the process of “*feature reset*” was explicitly provided in **Module 1** of **Section 3.2.1**, and all corresponding changes were highlighted in BLUE color. Moreover, in the revised manuscript, **Figure 3** was redrawn to clarify the relationship between the two maps before and after the “*feature reset*”. The detail information on the “*feature reset*” process was also further provided in **Supplementary Method S2**.

**Comment 7**

Major: - It seems that the main component of the architecture addressing the long-tail problem is the LSTM. This is mentioned in the Methods section but it is difficult to exactly understand the underlying rationale and again, its is difficult to relate what is written in the manuscript and what is contained in the cited references. Could the authors move this part to the introduction and improve clarity?

**Response to Comment 7**

Thanks a million for the valuable comments. We felt sorry that there might be some mis-leading statements in our original submission. Based on the performance of *AnnoPRO* and those multiple case studies provided in our manuscript, we have a strong feeling that the ability of our *AnnoPRO* in addressing the so-called *long-tail problem* does not solely come from the LSTM, which should be a collective contribution of multiple modules within our hybrid deep learning frameworks. In particularly, there are three consecutive modules. As shown in **Figure 2**, these modules included: ***M1***) the sequence-based multi-scale protein representation realizing the conversion of sequences to the *feature similarity*-based images (*ProMAP*) and *protein similarity*-based vectors (*ProSIM*). ***M2***) the dual-path protein encoding based on pre-training. Using *ProMAP* and *ProSIM* generated for all proteins, a dual-path encoding was constructed. ***M3***) the functional annotation by a LSTM-based decoding. The protein features pre-trained using the dual-path encoding layer in ***M2*** were concatenated and then fed into the LSTM to enable a multi-label annotation of proteins to 6,109 functional GO families using the hybrid deep learning. During the revision, *ablation* experiment was conducted to assess how the performances of *AnnoPRO* were affected by these key modules. As shown in **Supplementary Figure S1**, if the LSTM is made absent from the model, *AnnoPRO* will suffer from a substantial reduction in the annotation performance (-14.0~-19.4% for AUPRC; -5.0~-18.2% for F*_max_*), which represented the great contribution of LSTM in addressing *long-tail problem*. However, if another module named ‘*ProMAP*’ was deprived from our model, *AnnoPRO* will experience the much higher reduction (compared with LSTM) in its annotation performance (-16.7~-36.8% for AUPRC; -4.6~-22.4% for F*_max_*). All in all, *AnnoPRO*’s abilities to address the *long-tail problem* may originate from multiple perspectives, and all those modules work together to contribute to the good annotation performance of our newly constructed model. In our revised manuscript, a new paragraph was added at the beginning of **Section 2.1** and *ablation* experiments were explicitly described in the third paragraph of **Section 2.2**, to explaining the above problems (corresponding modifications were all highlighted in BLUE color).

**Comment 8**

Are the 11 neurons in the LSTM enforced to match the 11 levels in the GO hierarchy? Is the output a “flat” representation of the GO hierarchy, i.e. a 6109-dimensional vector with arbitrary order?

**Response to Comment 8**

Thanks a million for pointing out this problem. The number of neurons is one of the optimizable parameters in our model. We felt sorry that the number provided in our original submission was not correct, which should be corrected to 256. During the revision, we carefully double-checked all parameters adopted in model construction, and some of the representative ones were provided in **Section 3.2.2**, **Supplementary Table S3**, and **Supplementary Figure S6**. Furthermore, those outputs of the model are indeed ‘flat’ representation of the GO hierarchy, and the order of 6,109-dimensional vector was directly adopted from the family relation file ‘go.obo’ downloaded from the *Gene Ontology* database (*Genetics*. 224: iyad031, 2023).

**Comment 9**

Major: - I had a very strange experience with the web server. The description of the method did not match that reported in the manuscript. For instance, there was some mention of protein structure descriptors and of AlphaFold2 predictions. I queried P0DP23, human calmodulin, but the server did not execute. I queried an example case provided on the main page, and the server seemed to compute forever. I could not access the results. Moreover, it was written that I had consumed 2 out of 16 free submits, suggesting that the server is not accessible freely to all. Moreover, is the source code or the architecture available and trainable?

**Response to Comment 9**

Thanks for pointing out the problems of our web-server, which might be caused by the temporary network interruption between web interface and core calculation unit. Moreover, we agreed with the reviewer that the description of the methods online did not fully match with that provided in the original manuscript. During the revision, our online server was reconstructed, and four major optimizations were provided: (**1**) the description of the methods online completely matched with the content in our revised manuscript; (**2**) the network connection between the web interface and the core calculation unit was strengthened; (**3**) the sever is now made accessible freely to all; (**4**) all source codes for protein functional annotation using *AnnoPRO* were now available on GitHub (<https://github.com/idrblab/AnnoPRO>) and Zenodo (<https://zenodo.org/records/10012272>). The web-server realizing *AnnoPRO* prediction (<https://idrblab.org/annopro/>) is now accessible by all users, and a pypi package (<https://pypi.org/project/annopro/0.1rc2/>) is also provided.

**Comment 10**

Major: - The method on which the work is inspired, corresponding to ref 44 was published in 2016. Could the authors provide more elements to support the fact that it is state-of-the-art as of 2023. It is also very strange that this ref 44 is not mentioned in the Methods section.

**Response to Comment 10**

We would like to thank the reviewer for these valuable comments. The deep learning framework constructed in this study was inspired to some extent by a method applied for image classification (*IEEE Conf Comput Vis Pattern Recognit*. 2016: 2285, 2016), since both methods were designed to cope with their own *long-tail problem*. We agree with the reviewer 2 that it is inappropriate to say that the previous image classification method is state-of-the-art as of 2023. Therefore, in the revised manuscript, the corresponding statement was revised to:

“Since this framework was inspired, in part, by a method [33] used for image classification to cope with ‘*long-tail problem*’, *AnnoPRO* was expected to significantly improve the annotation performance for the GO families in the ‘*Tail Label Levels*’”

Corresponding modifications were made to the last paragraph of the **Background** section, which were all highlighted in BLUE. Moreover, in the revised manuscript, the corresponding reference [33] was also described and cited in the ***Module 2*** of **Materials and Methods** section.

**Comment 11**

Major: - Figure 2 and the method should be briefly described in the Results and Discussion section before the comparison with other methods. Currently, Figure 2 is introduced only with the ablation study. Please, clearly indicate M1, M2 and M3 on Figure 2. The use of multiple colors in different parts of the architecture makes the schema difficult to understand.

**Response to Comment 11**

Thanks a million for these very constructive suggestions. During the revision, a new **Section 2.1** was added at the beginning of **Results and Discussion** for briefly describing our newly proposed hybrid deep learning method and **Figure 2** (all corresponding modifications were highlighted in BLUE color). Moreover, **Figure 2** was modified by adding essential labels and optimizing color scheme. The newly added paragraph in **Section 2.1** was highlighted in BLUE color.

**Comment 12**

Major: - Could the authors define multimodality and describe explicitly in what way their framework is multimodal? 1D vectors and 2D matrices have already been used to represent proteins and given as combined inputs to deep neural nets. What is the novelty brought by this work in that respect?

**Response to Comment 12**

Thanks for the valuable questions. During the revision, we further elaborated the architecture of our *AnnoPRO* model, especially clarified those novel strategies proposed in this work for protein representation. Particularly, a hybrid deep learning framework was constructed to enable protein function annotation, which consisted of three consecutive modules. As provided in **Figure 2**, the ***Module M1*** proposed an innovative protein representation strategy of multiple scale that realized the conversion of all protein sequences to *feature similarity*-based images (*ProMAP*) and *protein similarity*-based vectors (*ProSIM*). Particularly, at *feature similarity* scale, the similarities among protein features were utilized to transform the ‘unordered’ vector of 1,484 protein features to an ‘ordered’ image-like representation; at *protein similarity* scale, the pair-wise similarities between any two proteins were used to transform the ‘independent’ vector of 1,484 protein features to a ‘globally-relevant’ vector of 92,120 dimensions. It is clear to see that these new strategies above are totally different from traditional 1D vector and 2D matrix. In other words, our new strategies are unique in capturing the intrinsic correlation among protein features, but also taking the global relevance among protein sequences into consideration. As a result, we would like to express our sincerely appreciation to reviewer 2 for offering us this opportunity to further elaborate the newly proposed representation strategy together with hybrid deep learning models. During the revision, the corresponding modifications were made to the end of **Background**, the beginning of **Section 2.1**, and the **Module 1** of **Section 3.2.1** (all changes were highlighted in BLUE color).

**Comment 13**

Major: - The description of the method lacks clarity. In particular, when describing the construction of the image using “U-Map”, the authors cite a publication about the Tanimoto index. In what way is that relevant? Where is the reference for “U-Map”? Did the authors use this method: L. McInnes, J. Healy, and J. Melville. Umap: Uniform manifold approximation and projection for dimension reduction, 2020. arXiv:1802.03426? To map the features in 2D according to their similarities? If yes, then the authors should discuss the limitations of U-Map. Lior Pachter's group has recently shown that UMAP produces arbitrary shapes in 2D and distort distances, which can severely bias and mislead interpretation.

**Response to Comment 13**

Thanks a million for pointing out these problems. Indeed, the previous citation is not appropriate. In the revised manuscript, the correct publication of ‘UMAP’ was cited, which is the same as the one provided by the reviewer. Many thanks! Moreover, we agree with the reviewer that there are reported limitations of UMAP. Therefore, during the revision, a discussion on its limitation and the way how the annotation performance was affected were explicitly described by adding a new paragraph at the end of **Section 2.2** (provided below). Modifications were highlighted in BLUE.

When realizing the image-like protein representation (as illustrated in **Figure 3**), there were two methods applied for *dimensionality reduction*, which included *uniform manifold approximation and projection* (UMAP) [36] and *principal component analysis* (PCA) [37]. UMAP was reported to produce arbitrary shapes in 2D and distort distances, which could be severely biased and lead to misinterpretation [43]. Since the image-like protein representation was novel and essential for *AnnoPRO*, it is needed to assess the contributions of UMAP and PCA to annotation performances. Herein, two models *AnnoPRO_UMAP_* and *AnnoPRO_PCA_* were thus constructed based on UMAP and PCA, respectively (the procedure for model construction and evaluation is described above). As shown in **Supplementary Figure S5**, the performances (assessed by F*_max_* and AUPRC) of these models are roughly the same across three GO classes (BP, CC, MF). Particularly, *AnnoPRO_UMAP_* gave a slightly better predictive performance compared with *AnnoPRO_PCA_* (+0.6~+1.9% for F*_max_*; +1.4~+2.1% for AUPRC). In sum, although concerns were raised about the limitations of UMAP [43], the performance evaluation conducted in this study showed that the application of different dimensionality reduction methods (UMAP *vs* PCA) might not significantly alter the performance. Therefore, both methods (UMAP and PCA) were integrated into the *AnnoPRO* software package (<https://pypi.org/project/annopro/0.1rc2/>) and the online server (<https://idrblab.org/annopro/>).

**Comment 14**

Major: - What were the training procedure used for the state-of-the-art ML-based methods the authors compare AnnoPRO with? Are they exactly the same for all methods? If yes, this should be stated more clearly. If not, this should be discussed.

**Response to Comment 14**

Thanks for asking. Yes, the training procedures for all studied methods including our *AnnoPRO* were identical. During the revision, a new **Supplementary Method S1** was offered to explicitly describe their procedures for model construction. Many thanks!

**Comment 15**

Were the proteins used to showcase the results, namely growth differentiation factors (GDF8 & GDF11) and heat shock proteins (HSPA1A & HSPA2) part of the training or validation set. Could the authors provide the link to the CAFA data used for training, validation and testing? The description of training/testing procedures for AnnoPRO and for the other methods should be described in the Material and Methods section.

**Response to Comment 15**

Thanks a million for your valuable comments. *First of all*, both the growth differentiation factors (GDFs) and heat shock proteins (HSPs) were not in either training or validation data, but adopted as the independent testing. *Second*, all training, validation and independent testing data together with source codes have been made available to all users, which could be freely downloaded from GitHub (<https://github.com/idrblab/AnnoPRO>) and web-server (<https://idrblab.org/annopro/>).

**Comment 16**

Minor: - There are several sentences that are almost copied-pasted in multiple places throughout the text, and English writing could be improved.

**Response to Comment 16**

Thanks a million for reminding us to remove the duplicated description among results, discussion and methods. During the revision, a careful review on our manuscript and the corresponding text editing were conducted to provide different descriptions among these sections. All corresponding modifications were highlighted in BLUE color in our revised manuscript. We really appreciated that you brought this issue to our attention during the peer-review process, and the latest version of our manuscript became much clearer compared with our previous submission. Thanks!

Moreover, thank you for pointing out the language issues. During the revision, we proof-read the entire manuscript, and improved the language by consulting our colleagues. The native language of our colleagues is English, and they are from either my research directions or school of foreign languages in my university. Based on their suggestion, modifications were made throughout the manuscript and highlighted in BLUE color. Thanks a million for your valuable comments.

**Comment 17**

Minor: - The feature similarity matrix does not encode similarities, but distances. Maybe rename it? How are the protein similarities computed?

**Response to Comment 17**

Thanks a million for the professional suggestions. During the revision, we renamed it to “*feature distance matrix*” (FDM) based on your valuable comment. Moreover, the way to compute protein similarities were explicitly described in the *Fourth* part of the **Module 1** in **Section 3.2.1** (all the modifications were highlighted in BLUE color). Many thanks!

**Comment 18**

Minor: - In the introduction, could the authors be a bit more specific about the limitations of SH-based methods? “They are severely dependent on the homology…” is kind of expected for this type of methods and does not bring much information.

**Response to Comment 18**

We would like to thank reviewer 2 for pointing out this problem. During the revision, a literature review was conducted to specify the limitation of SH-based methods. As described in a previous publication (*Genome Biol*. 18: 186, 2017), the accuracy of sequence alignments drops off rapidly in cases where the sequence identity falls below a certain critical point. Therefore, in the revised manuscript, the corresponding sentence in the **Background** section was modified to:

“SH-based strategy has long been used for protein function annotations [22], and many tools have been constructed (such as *BLAST* and *GoFDR*) [23, 24], but the accuracy of sequence alignments drops off rapidly in cases where the sequence identity/homology falls below certain critical point [25]”

All corresponding modifications were highlighted in BLUE color.

**Comment 19**

Minor: - Figure S3 and S4: red fonts and blocks. Correct to only red blocks? (The fonts are black)

**Response to Comment 19**

We would like to thank you for the suggestion. During the revision, those fonts were changed to black, and only the blocks were kept RED. Again, thanks a million for your professional supports on improving our manuscript. With my very best wishes!

**Responses to Reviewer 3**

**Comment 1**

This study describes an approach for the automated function prediction of proteins. The approach is composed of a representation scheme and a computational method. The authors claim that the described approach is particularly efficient for the proteins that has relatively small number of functional annotations. The results and the approach is of interest to the audience. I have a major comment and a few minor remarks.

**Response to Comment 1**

Thanks a million for your valuable comments. During the revision, we have made point-to-point response to each of your comment, and the corresponding modifications were made to the latest version of our manuscript (highlighted in BLUE) and the provided source codes.

**Comment 2**

Major comment: The authors should evaluate the performance of the approach on other datasets and compare it with those of the state-of-the-art methods. For example, the datasets indicated in the following article can be employed and the performances can be compared. Unsal, S., Atas, H., Albayrak, M. et al. Learning functional properties of proteins with language models. Nat Mach Intell 4, 227-245 (2022). <https://doi.org/10.1038/s42256-022-00457-9>

**Response to Comment 2**

We would like to thank the reviewer for the very constructive comments. During the revision, a literature review on that suggested publication (*Nature Mach Intell*. 4: 227, 2022) was conducted, and two benchmarks were collected from that pioneering study to further assess the effectiveness and stability of *AnnoPRO*. The ***first*** benchmark was named PROBE in the original study (*Nature Mach Intell*. 4: 227, 2022), which contained 20,421 unique human proteins of distinct sequences. Following the same criterion (using Oct 22, 2019 as a *cutoff date*) used in CAFA4 for partitioning data, all these proteins were partitioned to 18,058 proteins (adopted as ‘*Training*’ and ‘*Validation*’ datasets for model construction) and 2,363 proteins (adopted as *Independent Testing*). *AnnoPRO* and the SOTA methods (such as *DeepGOPlus* and *PFmulDL*; Since *NetGO3* did not provide its training codes, it could not be retrained and evaluated for comparison here) were then completely retrained using those partitioned data described above. As shown in **Table 6**, *AnnoPRO* achieved the best performance on all GO classes, when compared with other two models. Particularly, the F*_max_* and AUPRC of *AnnoPRO* were much higher (4.5~18.8% and 4.9~24.0%, respectively) than that of two other models, which validated its effectiveness and stability in function annotation.

The ***second*** dataset was entitled ‘ontology-based PFP benchmark’ in that pioneering publication (*Nature Mach Intell*. 4: 227, 2022), which consisted of 25 sub-datasets. As described in the ‘*table S5*’ of that study, a protein representation method named ‘ProtT5-XL’ gave the best-performance in most (16 out of 18) of the GO groups/categories, while the method called ‘ProtALBERT’ gave the best-performance in the remaining categories. Thus, it is of interest to compare the annotation performances among *AnnoPRO*, *DeepGOPlus*, *PFmulDL*, and best-performing methods (BPMs) under the 18 categories using the same sub-datasets and partition strategy (5-fold cross validation) as that of the original study. Their performances (assessed using F*_max_*, which was the same as the original study) under those 18 GO categories were provided separately in **Figure 6** based on BP, MF, and CC. As demonstrated, *AnnoPRO* gave the best performances in most (17 out of 18) GO categories, which further showed the effectiveness and stability of *AnnoPRO* in protein function annotation. It is necessary to emphasize that the performances of BPMs of the original study are generated by an SVM-based prediction model. If this model can be further optimized to the one that is well complementary to the studied protein representation method, it would be anticipated that the corresponding performance of functional annotation could be further elevated.

All in all, we would like to express our great appreciation to Reviewer 3 for the very constructive suggestions. Although it took plenty of GPU computing time, the revision based on your valuable comments further validated the effectiveness & stability of *AnnoPRO* on additional benchmarks, and compared *AnnoPRO* with those of the state-of-the-art methods. Modifications were made at the end of “**Results and Discussion**” by adding two new paragraphs (highlighted in BLUE), and one new table (**Table 6**) and another new figure (**Figure 6**) were added to the main text.

**Comment 3**

Minor: Pp3, lines 5-6. Please explain. “Compared with protein sequences, the acquirement of experimentally-validated protein functions is much more challenging …”

**Response to Comment 3**

We would like to thank you for providing us the opportunity to clarifying this particular statement in our original submission. The reason why this statement was provided here was to illustrate the extreme difficulty in the experimental annotation of protein functions (experimentally-validated protein function). As described in the previous publication (*Nature Mach Intell*. 3: 1050, 2021):

“Fewer than 1% of the available protein sequences are currently annotated with reliable information, and the gap between unannotated and annotated sequences is widening at an unprecedented rate. Traditional experimental approaches to determine protein function are usually expensive, time-consuming and provide low throughput”

We felt sorry for not providing clear clarification in our previous submission. During the revision, the corresponding statement was thus modified to (modifications were highlighted in BLUE):

“Compared with the accumulation of protein sequences, the experimental annotation of protein functions is much more challenging …”

All in all, we thank reviewer 3 for the valuable comment, which made the current version of our manuscript much more academically rigorous than our previous submission.

**Comment 4**

Minor: Pp3, lines 7-8. Please cite the reference for 90,000 proteins whose functions have been successfully annotated. Can you also check if this number is up to date? “So far, the functions of only 90 thousand proteins have been successfully annotated, …”

**Response to Comment 4**

We would like to thank the reviewer for these professional comments. In our original submission, the number 90,000 was adopted from a previous publication (*Nucleic Acids Res*. 49:W469, 2021), which greatly increased from the number of 66,000 reported by another study (*Genome Biol*. 20: 244, 2019). Such result indicated that the number of proteins of experimental function annotation kept growing over time. Therefore, during the revision, we conducted a careful literature review to check whether the number (90,000) described in our original submission was still up-to-date. As described in the latest release of UniProt (*Nucleic Acids Res*. 51: D523, 2023), about 100,000 proteins that were manually annotated based on experimental evidence, were collected to current UniProt. In other words, it is difficult to provide “up-to-date” statistics as the numbers may differ among different studies. Thus, in the revised paper, we modified the corresponding part to:

“So far, only a very small portion of protein sequences have been successfully annotated based on experimental evidence [4], …”, and citation [4] is *Nucleic Acids Res*. 51: D523, 2023.

All in all, we thank reviewer 3 for the valuable comment, which made the current version of our manuscript much more academically rigorous than our previous submission.

**Comment 5**

Minor: Pp4, line 13. Can you show how the method is capturing the intrinsic correlations among protein features? “method is unique in capturing the intrinsic correlations among protein features”

**Response to Comment 5**

Thanks a million for this very professional comment. As illustrated in **Figure 3*a*** and **Figure 3*b***, each protein sequence was first represented by a vector of 1,484 features. To capture the intrinsic correlation among the features, an image (named *ProMAP*) was generated for each protein using the techniques of ‘*similarity calculation*’, ‘*dimensionality reduction*’, and ‘*allocation algorithm*’ (detailed description on the way to generate *ProMAP* was provided in Section 3.2.1 of the revised manuscript). To test whether the correlations among protein features were successfully captured by *AnnoPRO*, ablation experiment was conducted. As illustrated in **Supplementary Figure S1**, if *AnnoPRO* is deprived of the strategy of *ProMAP* generation (indicated by ‘*No ProMAP*’), the *area under the precision-recall curve* (AUPRC) for predicting functional families in BP, CC and MF will decrease 24.2%, 36.8% and 16.7%, respectively. In other words, the strategy of *ProMAP* generation contributed substantially to the good performance of *AnnoPRO*, and this is the reason why we stated that our method captured the intrinsic correlations among protein features.

**2^nd^ round**

**Reviewer 1**

Authors have improved the readability of the paper and addressed my major concerns

**Reviewer 2**

The authors have satisfactorily addressed my concerns. the manuscript has improved a lot.
Out of curiosity, I have two additional questions :
1/ It is a bit surprising that the authors do not use any dropout. At least, this is not mentioned. Could the authors confirm that it is not necessary for the model to generalize to unseen proteins?
2/ when using PCA instead of UMAP, how much of the total variance do the chosen 2 first principal components explain?

**Reviewer 3**

I thank the authors for their responses and modifications in the manuscript. All of my comments and questions have been satisfactorily responded.

**Authors’ response**

Referee 2 asks that you comment on whether dropout is not needed for the model to generalize to unseen proteins, and mention how much variance is accounted for by the first two principal components. We also ask that you define ‘head label level’ and ‘tail label level’ where you first use them in the introduction, which will aid reader understanding.

Response to Comment 2

Thanks a million for these valuable comments from you and the reviewer.

First, two dropout layers were incorporated in AnnoPRO after the application of CNN and before the application of LSTM. This technique can effectively reduce the overfitting and enhanced the prediction of novel proteins. This information is provided in the main text and Table S3.

Second, when using PCA, the 2 first principal components accounted for 96.7% and 1.4% of the total variance, summing up to 98.1%. In other words, AnnoPRO can greatly reduce the data dimensionality and preserve most of the data information by using only the 2 first PCs.

Third, during the revision ‘head label level’ and ‘tail label level’ were defined in the second paragraph of the Background (highlighted in BLUE):
